# A single point mutation is sufficient to drive *syp*-dependent biofilm formation and promote colonization by *Vibrio fischeri*

**DOI:** 10.1128/jb.00131-25

**Published:** 2025-07-14

**Authors:** Brittany L. Fung, Elizabeth G. Musto, Linsey Kathure Mugambi, Madison L. Lange, Jovanka Tepavcevic, Karen L. Visick

**Affiliations:** 1 Department of Microbiology and Immunology, Loyola University Chicago, Maywood, Illinois, USA; 2 Department of Biological and Health Sciences, Wheaton College 8560 , Wheaton, Illinois, USA; Geisel School of Medicine at Dartmouth, Hanover, New Hampshire, USA

**Keywords:** HnoX, *Euprymna scolopes*, two-component regulators, biofilm, *Vibrio fischeri*

## Abstract

Symbiotic colonization by *Vibrio fischeri* relies on the *syp* gene cluster, which encodes proteins predicted to synthesize and export a polysaccharide, SYP, that functions in cell-cell adherence. In strain ES114, four two-component sensor kinase/phosphatases, including central regulator SypF and the nitric oxide/HnoX-controlled HahK, dictate the activities of two response regulators, SypG and SypE, which in turn control SYP production. Here, we report that a single nucleotide change (C/A) upstream of the *hnoX-hahK* operon caused a substantial 80-fold increase in its transcription. While a search for negative regulators yielded Zur (zinc uptake regulator), loss of Zur only modestly (approximately threefold) increased transcription. We found instead that the C/A change engendered a new transcriptional start site. Furthermore, the C/A change was sufficient to robustly promote *syp*-dependent biofilm formation dependent on HahK and SypG but only partially dependent on the central regulator SypF. Rather, the residual biofilm formation in the absence of SypF relied on the luminescence regulator LuxU. Consistent with its ability to produce *syp*-dependent biofilms, a Δ*sypF* mutant that carried the C/A-*hahK* allele outcompeted its Δ*sypF* parent for squid colonization. Finally, bioinformatic analyses of the *hnoX* promoter region in various *V. fischeri* isolates revealed that most contained G or C nucleotides lacking in ES114, indicating an evolutionary divergence between different isolates. Together, these findings uncover the ability of HahK to signal through both SypF and LuxU to induce *syp*-dependent biofilm formation and host colonization, thus advancing our understanding of the regulators that control *syp*-dependent biofilm formation by *V. fischeri*.

**IMPORTANCE:**

Biofilms promote the attachment of bacteria to each other and to surfaces. For *Vibrio fischeri*, biofilm formation dependent on the symbiosis polysaccharide (*syp*) locus promotes colonization of its symbiotic host. Multiple two-component regulators, including the central sensor kinase SypF and nitric oxide/HnoX-controlled sensor kinase HahK, induce SYP production. Here, we identify a C/A change in the *hnoX-hahK* regulatory region that substantially increases its transcription and SYP-dependent biofilm formation. We further determined that HahK signals through both SypF and the luminescence regulator LuxU to promote biofilm formation and host colonization. Our findings thus provide insight into the regulatory crossover between two major pathways, quorum sensing-controlled luminescence and biofilm formation, in *V. fischeri*.

## INTRODUCTION

Bacteria promote their attachment to each other and to surfaces by secreting polysaccharides and other molecules into the environment to form protective assemblages known as biofilms (1, 2). Given the metabolic cost of synthesizing and secreting these biofilm-promoting substances, it is not a surprise that bacteria can exert intensive control over these processes. In many cases, bacteria use two-component regulators to sense the environment and appropriately induce or inhibit gene transcription.

**Fig 1:**
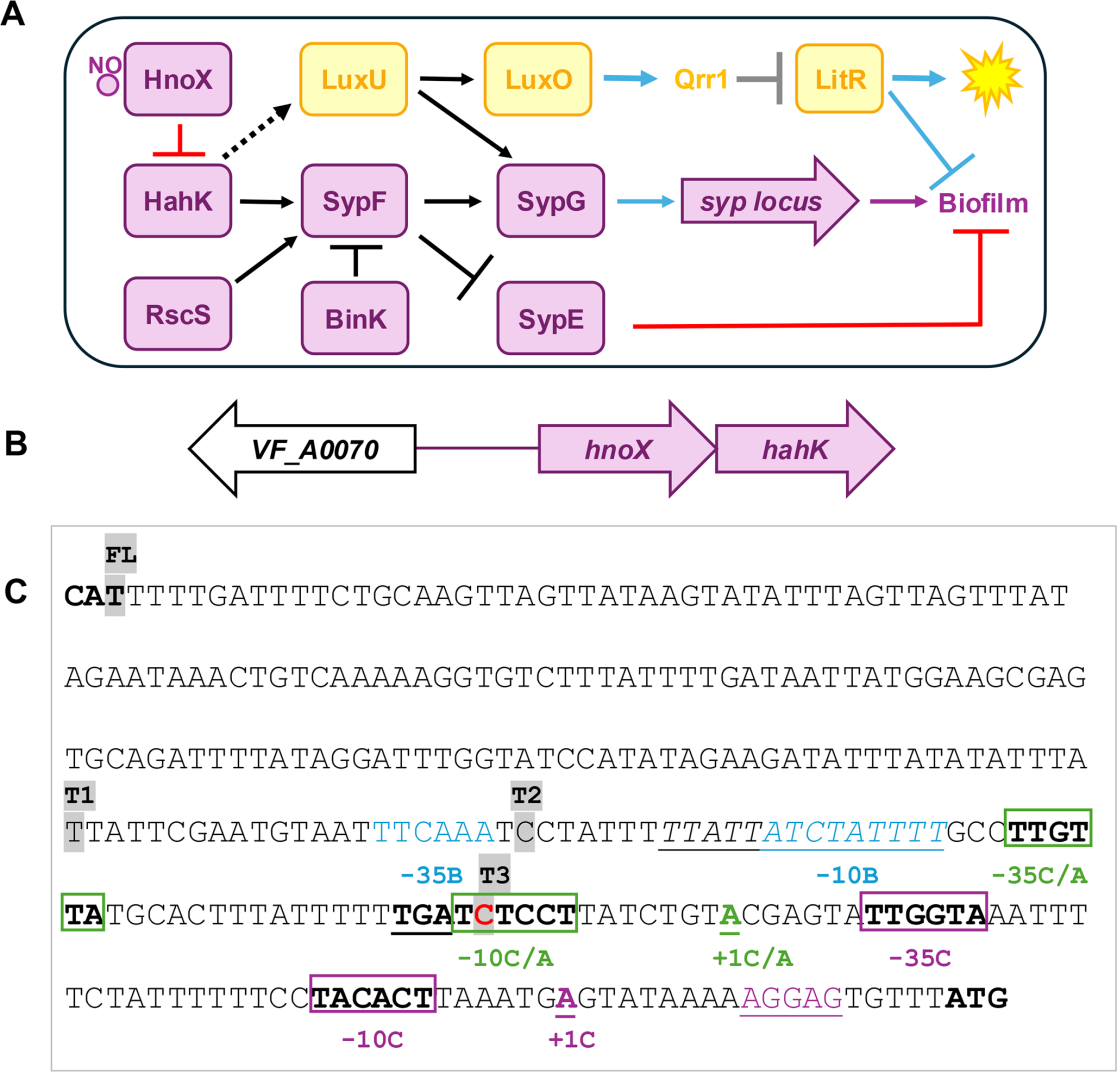
Regulation of and by HahK. (**A**) Model for regulatory control over biofilm formation (purple) and a portion of the luminescence pathway (yellow) in *V. fischeri*. Nitric-oxide (NO)-binding protein HnoX inhibits the activity of HahK, a positive regulator of *syp* transcription that contributes to the activation of sensor kinase SypF. SypF is also positively controlled by RscS and negatively controlled by BinK. In turn, SypF activates SypG, the direct transcriptional regulator of the *syp* locus, and inhibits SypE to induce *syp*-dependent biofilm formation. Proteins encoded by the *syp* locus synthesize, modify, and export SYP polysaccharide, an important component of *V. fischeri* biofilms. In the luminescence pathway, LuxU controls the activation of LuxO, which promotes transcription of the sRNA Qrr1. Qrr1 inhibits LitR production. LitR promotes light production and, through partially characterized pathways, inhibits biofilm formation. In this work, a previously unknown connection between HahK and LuxU was established in *V. fischeri* (dashed line). Connections in black denote phosphatase/kinase activity, gray denote post-transcriptional interactions, blue denote transcriptional interactions, and red denote an unknown mechanism. (**B**) The *hnoX-hahK* operon is transcribed divergently from *VF_A0070*, with 289 bp between the two ATG start codons. (**C**) The sequence between (and including the two ATG start codons, in bold) *VF_A0070* and *hnoX* is depicted. The BPROM-predicted promoter sequences are shown in blue text with the letter “B” following the annotations. The C base that was mutated in our study is shown in bold and in red. The +1 transcriptional start sites identified by 5' rapid amplification of cDNA ends (RACE) in the wild-type (WT) strain and in a strain that carries the C/A change are bolded and underlined, indicated with “+1C” in purple and “+1 C/A” in green text, respectively. Predicted promoters stemming from the 5' RACE results are indicated by purple and green boxes, respectively, with the −10 and −35 designations. For the promoter stemming from the C/A change, two additional elements are possible: a TGx extended −10 (underlined) sequence and an AT-rich region upstream of the −35 (italicized) that could serve as an up-element. The putative ribosome-binding site is indicated by the purple underlined text. The starting points of promoter-*lacZ* fusions are indicated by gray highlighting and text indicating full-length (FL), truncation-1 (T1), truncation-2 (T2), and truncation-3 (T3).

For the symbiont *Vibrio fischeri*, genes known to be important for *in vitro* biofilm formation are key for the assembly of bacterial aggregates on the surface of the symbiotic light organ of the squid *Euprymna scolopes*; these aggregates, in turn, promote efficient host colonization (3–7). The best-studied symbiotic strain of *V. fischeri*, ES114, produces a modest, but important, symbiotic aggregate dependent on the 18-gene *syp* locus (3, 5). The *syp* genes encode proteins that control the production, modification, and secretion of a putative polysaccharide, SYP, that facilitates cell-cell interactions leading to cohesive biofilm formation (3, 5–8). Until recently, the conditions under which ES114 could form *syp*-dependent biofilms in laboratory culture were unknown (9). Instead, cohesive biofilms dependent on *syp* were generated only upon overproduction of positive regulators and/or disruption of negative regulators (4, 10, 11). Despite this limitation, a complex network of regulators that control transcription and post-transcriptional processes leading to biofilm formation has been uncovered (reviewed in reference [12]). Of note, a central sensor kinase/phosphatase, SypF, is thought to integrate sensory transduction information from multiple two-component sensor kinases, then transmit that information to two response regulators, SypG and SypE, that control SYP production at transcriptional and post-transcriptional levels, respectively (4, 6, 8, 10, 11, 13) (Fig. 1A).

Current data support a model in which activating sensor kinases RscS and HahK promote phosphorylation of SypF, while the inhibitory sensor kinase BinK likely promotes dephosphorylation (Fig. 1A) (4, 8, 11, 13–15). Overproduction of RscS results in *syp*-dependent biofilm phenotypes such as wrinkled colonies on plates and pellicles under static liquid conditions and, clumps, strings, and rings under shaking culture conditions with the addition of the inducing signal calcium (4, 13). Similar phenotypes can be observed upon overproduction of HahK or of an active allele of SypF or with disruption of the gene for the inhibitory sensor kinase/phosphatase BinK (8, 11, 14, 16). *syp*-dependent biofilms are also formed upon overproduction of the transcription factor SypG when strains also lack the inhibitory protein, SypE (10).

While little is known about the signals controlling RscS and SypF, the activity of HahK is negatively controlled by the nitric oxide (NO)-sensing protein HnoX (17); the genes for the two proteins form an operon, *hnoX-hahK* (18, 19) (Fig. 1B). Squid-derived NO acts as a specificity factor for *V. fischeri* during colonization, and strains that lack *hnoX* or *hahK* exhibit increased and decreased colonization efficiencies, respectively (17, 20). *In vitro*, exposure to NO inhibits *V. fischeri* biofilm formation in a manner that depends on HnoX (17, 21). HnoX inhibits the activity of HahK through an unknown mechanism, thereby diminishing or preventing biofilm formation (21).

In addition to these major regulators, other factors contribute to biofilm formation by *V. fischeri*. For example, multiple studies have linked *syp*-dependent biofilm formation to another aspect of *V. fischeri* physiology, quorum sensing-regulated bioluminescence (22–24). For example, the histidine phosphotransferase, LuxU, which indirectly controls the luminescence-producing *lux* genes (25, 26), also contributes to control over biofilm formation via SypG (22). Furthermore, the quorum sensing-controlled regulator LitR influences the timing of architecture development of *syp*-dependent biofilms (27, 28) (Fig. 1A). The interconnectedness of the various *V. fischeri* pathways adds complexity to our understanding of their regulatory mechanisms.

Here, we expand our understanding of the HahK arm of the pathway through our discovery of a single, specific base in the upstream regulatory region that, when changed from C to A (C/A), substantially enhanced transcription of the *hnoX-hahK* operon, leading to induction of *syp*-dependent biofilm formation, in part via SypF. Our subsequent exploration yielded additional findings, including the identification of LuxU as a second target of HahK activity. Together, our study provides new insights into factors that feed into the control over *syp*-dependent biofilm formation by *V. fischeri*.

## RESULTS

### Bypass suppressor maps to *hnoX* promoter region

**Fig 2:**
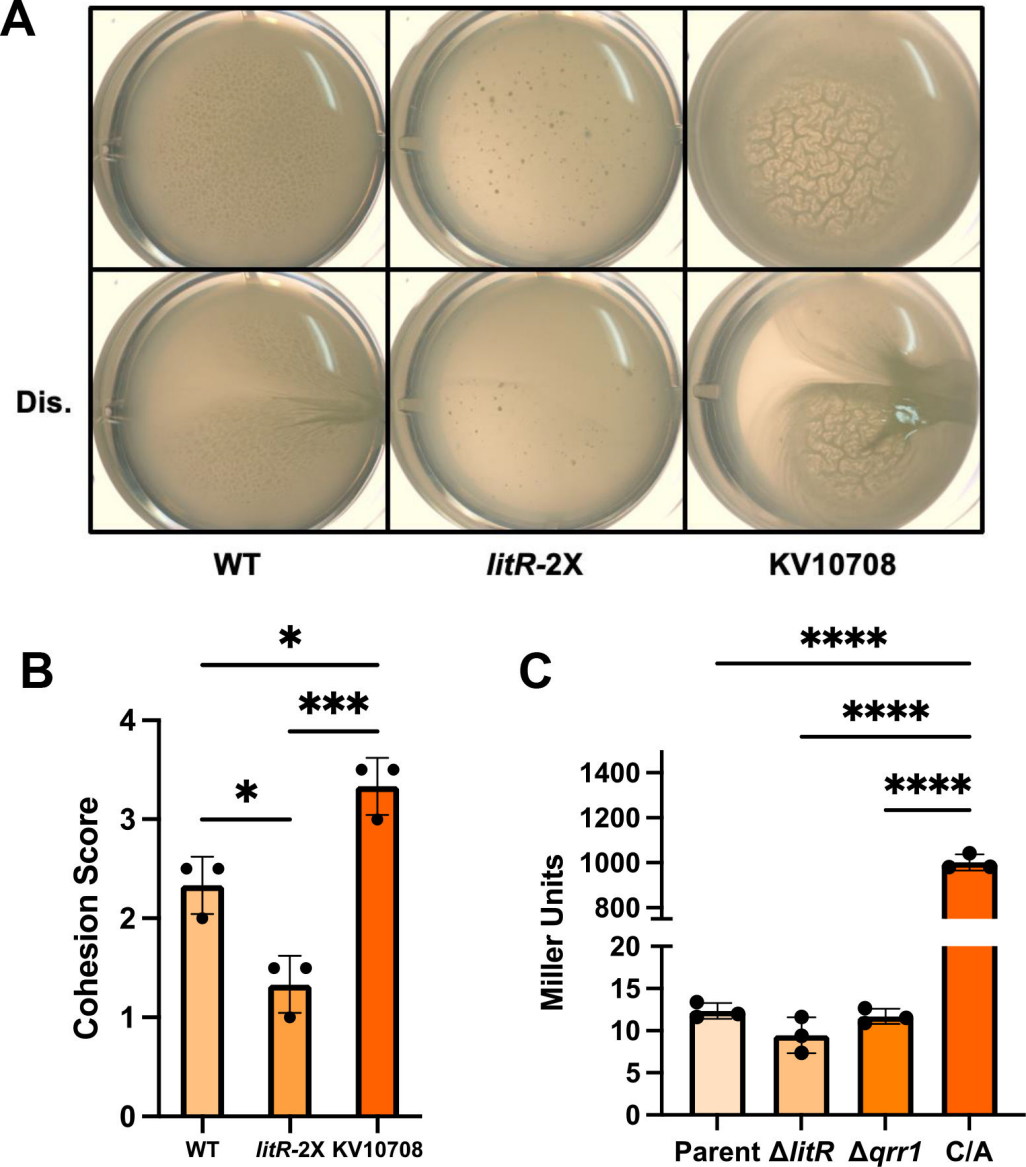
A point mutation in the *hnoX* promoter region increases pellicle production of a *litR* overexpressing strain. (**A**) Representative images of pellicles produced by wild-type (WT) strain ES114, *litR*-overexpressing strain KV10050 (*litR-*2X), and a derivative of KV10050 (KV10708) that contains a C/A change 73 bp upstream of the ATG start codon of *hnoX*. Cells were grown statically for 72 h in LBS +10 mM Ca^2+^. Top, undisturbed; bottom, disturbed with a toothpick (Dis.). (**B**) Biofilm cohesion was quantified from images similar to and including those shown in panel A as described in “Materials and Methods.” Pellicles were scored on a scale of 1–4, with 1 being the least amount of biofilm and 4 being the most sticky/cohesive. *, *P* ≤ 0.05; ***, *P* ≤ 0.001. Error bars represent SD. (**C**) β-galactosidase activity measurements for strains containing P*hnoX-lacZ* or, in one case, P*hnoX*-C/A-*lacZ*, as follows: P*hnoX-lacZ* (parent strain [parent]; BF450); P*hnoX-lacZ* Δ*litR* (Δ*litR*; BF451); P*hnoX-lacZ* Δ*qrr1* (Δ*qrr1*; KV10666); P*hnoX*-C/A-*lacZ* (C/A; BF478). All strains also contain a Δ*sypQ* mutation to prevent biofilm formation. Cells were grown with shaking for 22 h in LBS +10 mM Ca^2+^. ****, *P* < 0.0001. Error bars represent SD.

In related work seeking to determine how the quorum sensing regulator LitR inhibits biofilm formation, we found that loss of LitR from *V. fischeri* strain ES114 substantially enhanced pellicle formation of cells grown statically in the complex medium LBS that contained 10 mM calcium chloride (LBS-Ca) (29). Correspondingly, strain KV10050, which carries a second copy of *litR* in the genome (*litR-*2X), produced weaker pellicles, as observed by a general lack of cohesion (stickiness), than its parent strain ES114 (Fig. 2A and B), presumably due to increased LitR levels. Cohesion was quantified by scoring deidentified representative pellicle images on a scale of 1–4, with 4 being the stickiest. To identify factors that function downstream of LitR, we enriched for suppressor mutants of KV10050 that could form biofilms despite the activity of LitR by growing this strain statically in LBS-Ca, collecting cells from the surface of the culture, and reinoculating new media. Ultimately, we isolated mutants that were competent to form a pellicle. We report here the characterization of one suppressor mutant, KV10708, that produced visibly more robust pellicles under these conditions (Fig. 2A and B).

Whole-genome sequencing revealed that KV10708 contained a mutation within the regulatory region upstream of the *hnoX-hahK* operon (Fig. 1B and C). This mutation was of note because these genes encode negative and positive regulators, respectively, of the *syp* locus, which controls host-relevant biofilm formation by *V. fischeri* (Fig. 1A). Specifically, a C to A (C/A) mutation (shown in red-colored text in Fig. 1C) was positioned 73 bp upstream of the predicted ATG start codon of *hnoX*.

To determine if the C/A mutation impacted transcription of the *hnoX* gene, we fused the *hnoX* regulatory region upstream of a promoterless *lacZ* gene (P*hnoX-lacZ*) at a benign location in the *V. fischeri* genome (between *yeiR* and *glmS* [30]) and estimated transcription using β-galactosidase assays. A derivative of the reporter strain that carried the C/A point mutation produced units that were over 80-fold higher than that of the reporter strain carrying the wild-type (WT) *hnoX* regulatory region (Fig. 2C). These data support the conclusion that this single C/A mutation increases transcription of the *hnoX-hahK* operon.

Because the C/A mutation was initially generated in a *litR-*2X background, we asked if LitR could control the transcription of *hnoX*. However, similar levels of β-galactosidase activity were generated by the *hnoX* promoter reporter regardless of whether the strain background carried a deletion of *litR* or of the gene for its negative regulator, Qrr1 (Fig. 2C). Thus, we conclude that LitR does not control the transcription of *hnoX* and is unlikely to exert its negative effect on biofilm formation via *hnoX-hahK*. As a result, we shifted our focus away from probing the role of LitR in controlling biofilm formation (29, 31). Instead, given the importance of *hnoX* and *hahK* in the control of biofilm formation and squid colonization (11, 17, 21) and the relative lack of information about transcriptional control over these genes in *V. fischeri*, we sought to understand the consequences of the C/A mutation on *hnoX* transcription and on biofilm formation.

### Zur inhibits *hnoX* transcription

We used BPROM (32) to predict the position of the *hnoX* promoter. This software program identified a possible promoter, with putative −10 sequences centered about 32 bp upstream of the C/A change (blue text labeled −10B and −35B in Fig. 1C). Because the position of the C/A mutation relative to the BPROM-predicted promoter had the potential to affect the binding site of a repressor, we next sought to identify a regulator(s) that could control transcription of the *hnoX-hahK* operon by performing transposon (Tn) mutagenesis. We used a strain that carried the WT regulatory region upstream of a promoterless *lacZ* gene and screened mutants for a darker blue color on plates that contained X-gal. In multiple independent screens, we isolated transposon insertions in *VF_0306*. BLASTP (33, 34) analysis of the *V. fischeri* protein encoded by *VF_0306* revealed 56% identity and 72% similarity to the *Vibrio cholerae* El Tor strain N16961 Zur (zinc uptake regulator) protein, which has been recently characterized (35, 36). In *V. cholerae*, as well as many other bacteria (37–41), Zur functions to inhibit or activate genes in response to zinc.

**Fig 3:**
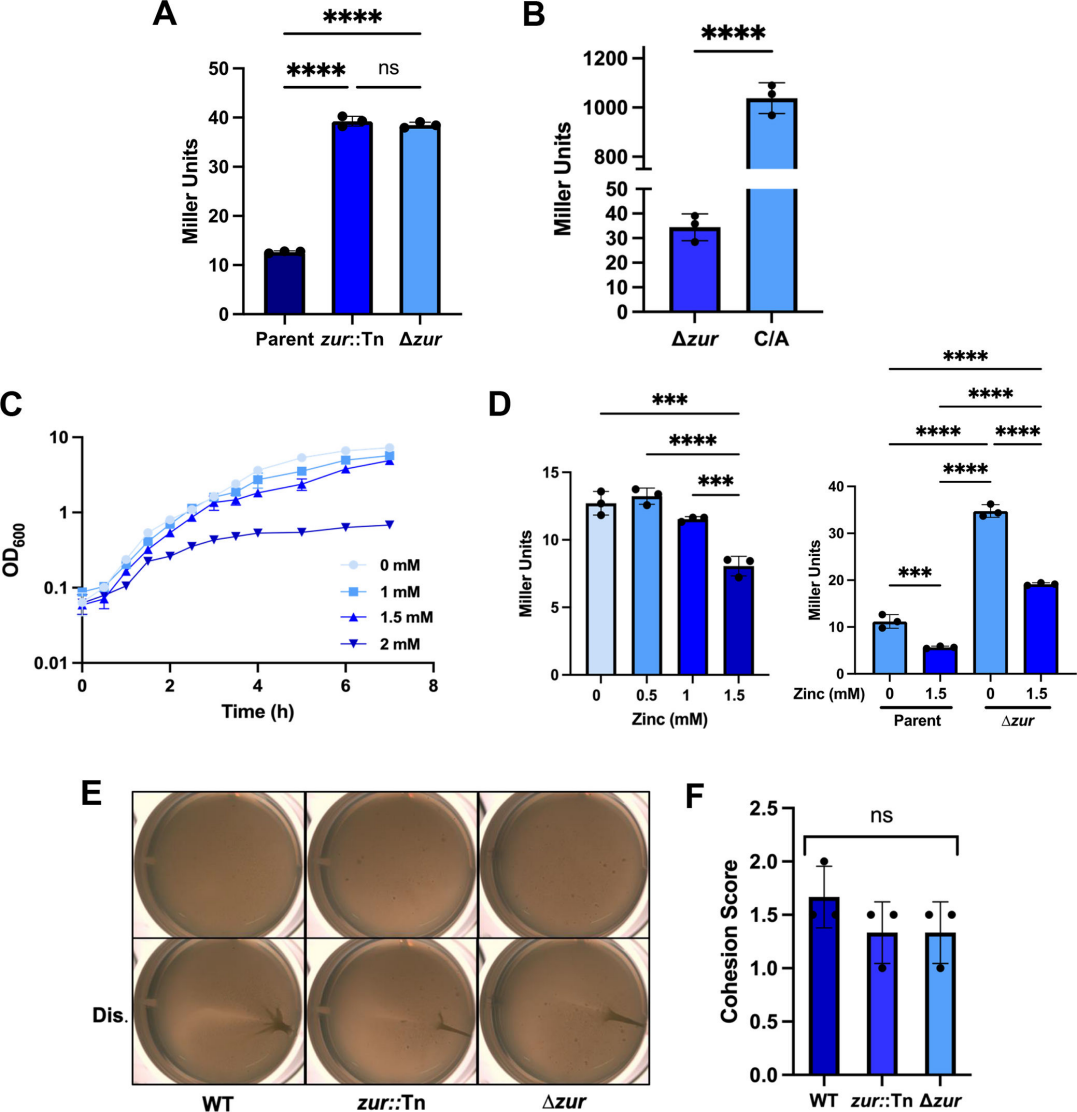
Zur inhibits transcription from the *hnoX* promoter. (**A**) β-galactosidase activity measurements for strains containing P*hnoX-lacZ* with an intact *zur* gene (parent; BF450), with the *zur* gene disrupted by transposon insertion (*zur*::Tn; KV10696) or with *zur* deleted (Δ*zur*; KV10668). Cells were grown for 22 h in LBS +10 mM Ca^2+^. All strains contain a Δ*sypQ* mutation to prevent biofilm formation during the experiment. ****, *P* < 0.0001; ns, not significant. Error bars represent SD. (**B**) Comparison of β-galactosidase activities for the Δ*zur* mutant that carries P*hnoX-lacZ* (Δ*zur*; KV10668) and the strain that carries P*hnoX*-C/A-*lacZ* (C/A; BF478), both of which carry the Δ*sypQ* mutation. Same experimental conditions as A. ****, *P* < 0.0001. Error bars represent SD. (**C**) Growth over time of strain ES114 in LBS containing different amounts of ZnCl_2_ (0–2 mM, as indicated in the key) at 24°C with shaking. Error bars represent SD. Some error bars cannot be seen as they are smaller than the size of the symbol. (D, left) β-galactosidase activity measurements for the P*hnoX-lacZ* and ∆*sypQ* (to prevent biofilm formation) containing strain BF450 grown in different concentrations of ZnCl_2_ (0–1.5 mM). Cells were grown for 22 h in LBS + respective amounts of ZnCl_2_. ****, *P* < 0.0001; ***, *P* ≤ 0.001. Error bars represent SD. (D, right) β-galactosidase activity measurements for the P*hnoX-lacZ* containing strain (parent; BF450) and the ∆*zur* mutant (KV10668) grown in 0 or 1.5 mM ZnCl_2_. Strains also carried a ∆*sypQ* mutation to prevent biofilm formation. Cells were grown for 22 h in LBS + respective amounts of ZnCl_2_. ****, *P* < 0.0001; ***, *P* ≤ 0.001. Error bars represent SD. (**E**) Representative pellicle images of ES114 (WT), *zur*::Tn (KV10528), or Δz*ur* (KV10552) strains. Cells were grown statically for 72 h in LBS + 10 mM Ca^2+^. Top, undisturbed; bottom, disturbed with a toothpick (Dis.). (**F**) Biofilm cohesion scores of pellicles similar to and including those shown in panel E. ns, not significant. Error bars represent SD.

To confirm the role of *zur* in controlling the transcription of *hnoX-hahK*, we deleted *zur* from the P*hnoX-lacZ* reporter strain. Both the newly generated *zur* deletion and a representative Tn insertion in *zur* caused an increase (~3×) in β-galactosidase activity from the P*hnoX-lacZ* fusion (Fig. 3A). These data support the conclusion that Zur negatively regulates *hnoX* transcription.

However, the increase in transcription caused by the loss of Zur, while significant, was much less than that caused by the C/A point mutation (Fig. 3B). We thus wondered if *hnoX* transcription would be altered by the addition of zinc to the medium. We first assessed the relative toxicity of zinc on the growth of WT strain ES114 under shaking conditions. We found that a zinc concentration of 2 mM substantially diminished growth, while concentrations of 1.0 and 1.5 mM exerted little to no effect (Fig. 3C). *hnoX* transcription was diminished when the reporter strain was grown in the presence of 1.0 mM or 1.5 mM zinc, but this effect was independent of Zur (Fig. 3D). Furthermore, disruption of *zur* did not significantly impact pellicle formation by an otherwise WT strain (Fig. 3E and F). We conclude that, while Zur and zinc decrease transcription of the *hnoX* promoter, Zur is not a major biofilm inhibitor under the conditions of our pellicle assay.

### C/A change generates new promoter

**Fig 4:**
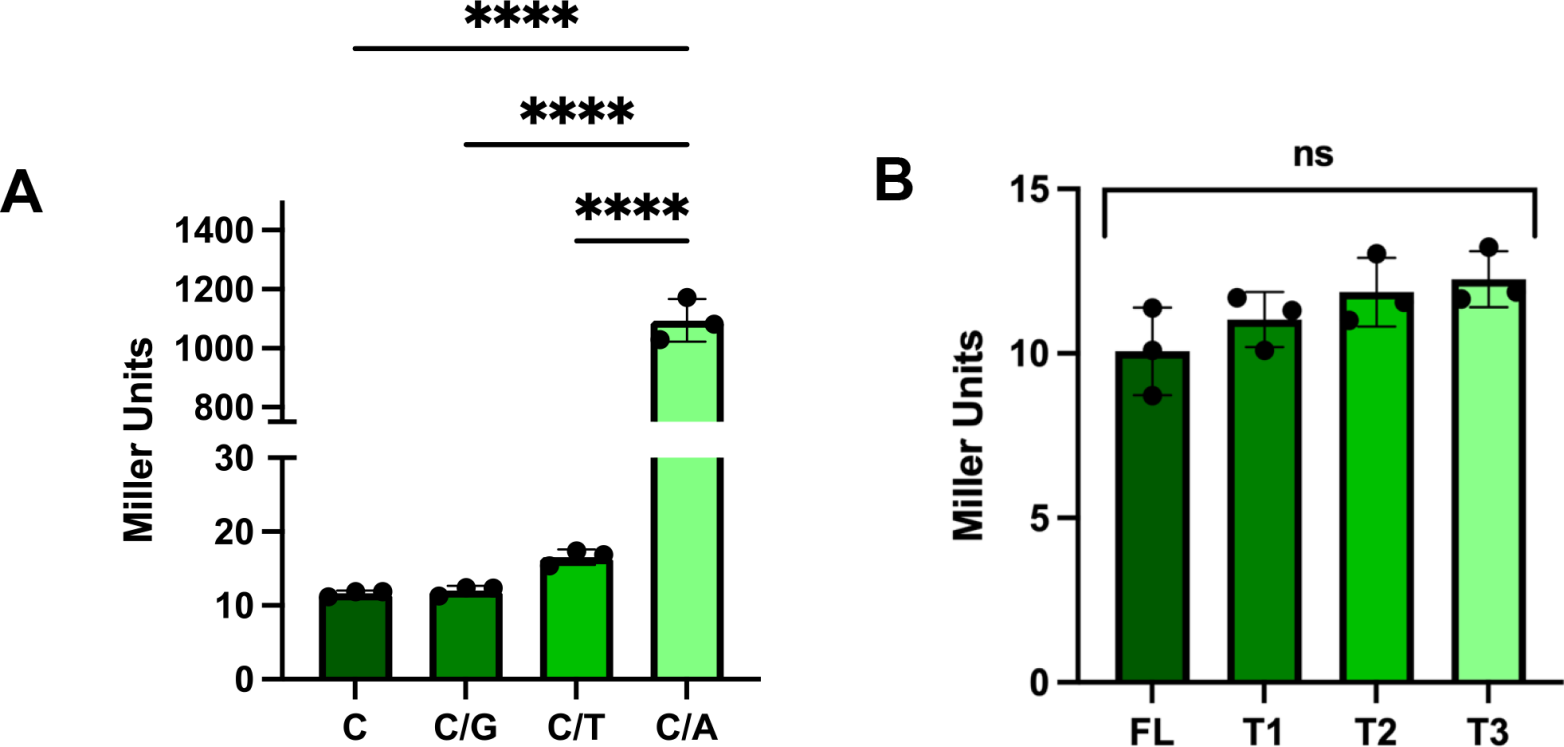
Neither other point mutations nor 5' truncation of the *hnoX* promoter region enhances transcription. (**A**) β-galactosidase activity of strains containing P*hnoX-lacZ* and, in the position 73 nucleotide upstream of the ATG start codon, the original nucleotide base C (C; BF450) or base changes to G (C/G; KV10645), T (C/T; KV10646), or to A (C/A; BF478). All strains contain a Δ*sypQ* mutation to prevent biofilm formation. Cells were grown for 22 h in LBS + 10 mM Ca^2+^. ****, *P* < 0.0001. Error bars represent SD. (**B**) β-galactosidase activity of strains containing 5' deletions in the *hnoX* promoter region as indicated in Fig. 1C. Promoter regions were full length (FL; BF450) or carried truncation 1 (T1; KV10648), truncation 2 (T2; KV10649), or truncation 3 (T3; KV10772). All strains contain a Δ*sypQ* mutation to prevent biofilm formation during the experiment. Cells were grown for 22 h in LBS + 10 mM Ca^2+^. ns, not significant. Error bars represent SD.

Because our Tn mutagenesis screens had not yielded leads other than *zur*, we turned our attention to more closely evaluating the *hnoX* promoter region. We first asked if the increased *hnoX* transcription was specific to the C/A change, or if other changes (C/G or C/T) would cause similar increases in transcription. However, transcription was not increased or otherwise impacted by the other two changes at this position (Fig. 4A). These data indicate that the C/A change makes a specific impact on the transcription of the *hnoX-hahK* operon.

To confirm or refute the tentative promoter identified using BPROM (32) (blue text in Fig. 1C), we constructed 5' deletions of the *hnoX* regulatory region in the context of the *lacZ* reporter (5' ends indicated by gray highlighting in Fig. 1C). Transcription was not impacted when we deleted sequences upstream of the predicted promoter (T1), through the predicted −35 sequence (T2), or through entire predicted promoter (T3; Fig. 4B). These data indicate that BPROM did not accurately predict the promoter.

Thus, we used 5' rapid amplification of cDNA ends (RACE) to identify the transcriptional start site and determine the position of the promoter. When we performed this experiment with the WT strain and with the Δ*zur* mutant, the start site of transcription was positioned 19 bp upstream of the ATG start codon—downstream from the C/A change and much closer to the coding sequence than predicted by BPROM (bolded purple text labeled +1C, −10C, and −35C in Fig. 1C; Fig. S1).

To begin to understand the consequences of the C/A change, we also used 5' RACE to determine the start site of transcription in a strain (KV10620) engineered to carry this mutation upstream of the *hnoX-hahK* operon inserted in the same benign position between *yeiR* and *glmS* as described earlier. In this strain, the start site of *hnoX-hahK* transcription was further upstream—61 bp upstream of the ATG start codon (bolded/underlined green text labeled +1C/A in Fig. 1C; Fig. S1). This transcription start site is positioned downstream of a putative −10 sequence that includes the C/A point mutation, TATCCT (rather than TCTCCT of the parent) (boxed in green, bolded, and labeled -10C/A in Fig. 1C). Just upstream is a TGn sequence that could serve as an extended −10 region, which permits additional sites of interaction between the RNA polymerase complex and the DNA (42–45). Further upstream and appropriately positioned is a sequence, TTGTTA, that could serve as the −35 element (boxed in green, bolded, and labeled -35C/A in Fig. 1C). In addition, upstream of the putative −35 lies an AT-rich sequence that may serve as an up-element (46–48) (italicized in Fig. 1C). These data support the conclusion that the C/A change generated an alternative promoter.

### C/A mutation promotes biofilm formation

**Fig 5:**
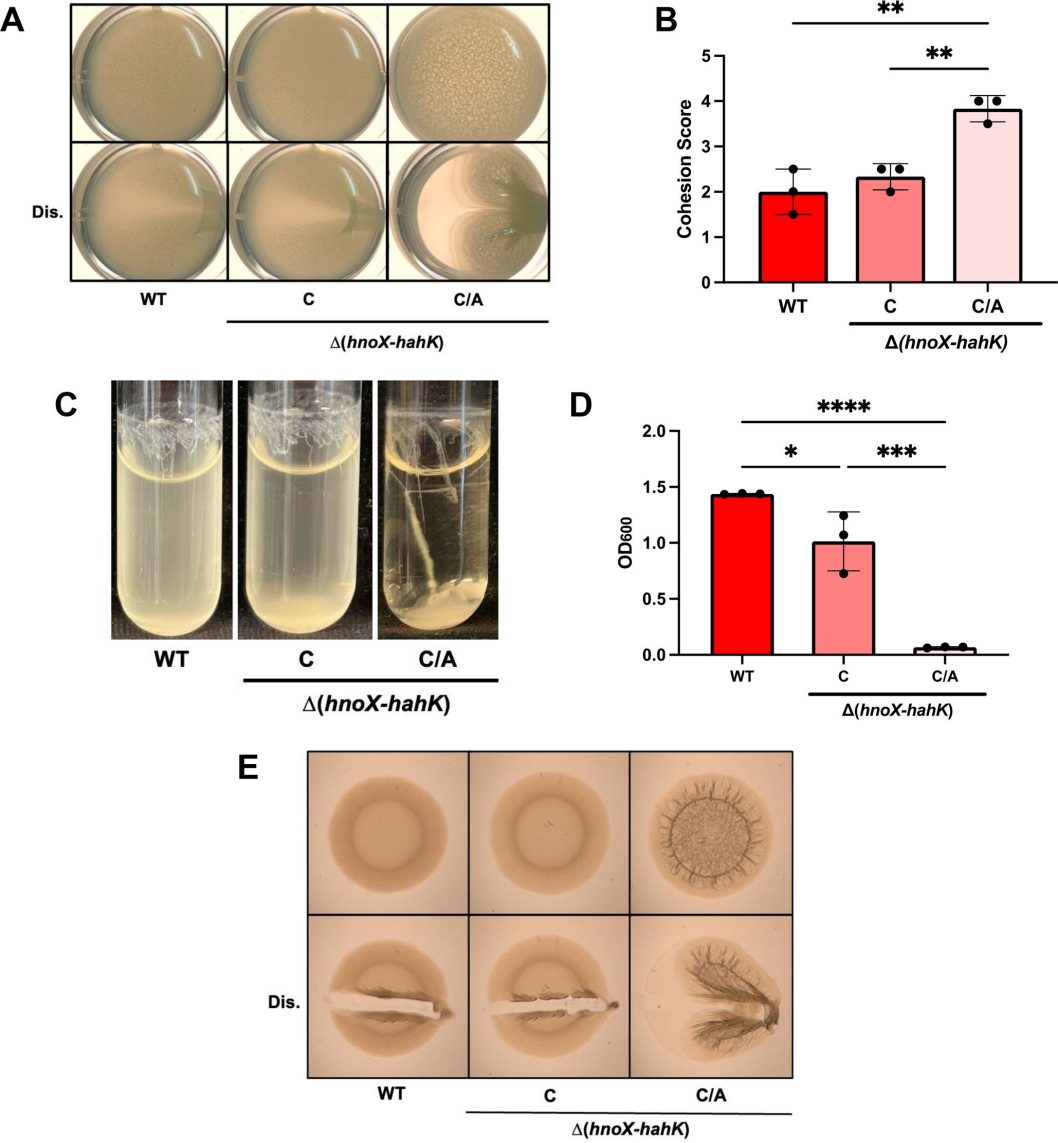
An *hnoX* promoter mutation increases biofilm formation under shaking liquid and plate conditions. For all panels, the strains tested were ES114 (WT) and two Δ(*hnoX-hahK*) derivatives that contained, at a non-native locus, either the *hnoX-hahK* operon (C; KV10513) or a derivative with the C/A change upstream (C/A; KV10620). (**A**) Representative pellicle images of strains grown statically for 72 h in LBS + 10 mM Ca^2+^ at 24°C. Top, undisturbed; bottom, disturbed with a toothpick (Dis.). (**B**) Biofilm cohesion scores of pellicles similar to and including those shown in panel A. **, *P* ≤ 0.01. Error bars represent SD. (**C**) Representative images of biofilms formed under shaking conditions. Cells were grown for 24 h at 24°C in tTBS +10 mM Ca^2+^. (**D**) OD_600_ measurements of the liquid surrounding the biofilms similar to and including those shown in panel C. *, *P* ≤ 0.05; ***, *P* ≤ 0.001; ****, *P* < 0.0001. Error bars represent SD. (**E**) Representative images of the biofilm formed on solid agar LBS plates containing 10 mM Ca^2+^ and incubated for 72 h at 24°C. Top, undisturbed; bottom, disturbed with a toothpick (Dis.).

We next evaluated the impact of the C/A point mutation on biofilm formation by generating and comparing a suite of strains that contained the *hnoX-hahK* operon, with and without the C/A change, at the same benign location in the chromosome that we used for the 5' RACE experiment. A strain that carried the unmutated locus as the only copy in the genome produced pellicles similar to its parent (Δ(*hnoX-hahK*)) and to the WT strain (Fig. 5A and B, Fig. S2). In contrast, a derivative of this strain that carried the upstream C/A mutation produced robust pellicles with striking architecture similar to the original suppressor mutant (KV10708; compare Fig. 5A with Fig. 2A). These data confirm that the single C/A mutation upstream of the *hnoX-hahK* operon is sufficient to promote biofilm formation.

We next asked if the C/A change promoted other types of biofilms. Specifically, we tested its ability to promote biofilms in shaking liquid culture and/or on plates. Indeed, the strain that carried the C/A change was able to produce enhanced biofilms under both conditions (Fig. 5C–E). These results suggest that this single point mutation is sufficient to drive robust biofilm formation under a variety of conditions.

We asked if the enhanced biofilms caused by the C/A change depended on calcium, a known inducing signal (11), and observed two levels of dependence. With respect to pellicle production, a lack of calcium diminished architecture but did not alter pellicle cohesion (Fig. S3A and B). In contrast, and consistent with other work (11), no biofilms formed under shaking liquid cultures in the absence of calcium (Fig. S3C and D). Similarly, on plates, the C/A mutant produced robust wrinkled colonies in the presence of calcium but little stickiness and no architecture in the absence of calcium (Fig. S3E). These results underscore the complexity of these different biofilm phenotypes and the involvement of additional regulatory inputs.

### HnoX diminishes the biofilm-promoting activity of HahK

**Fig 6:**
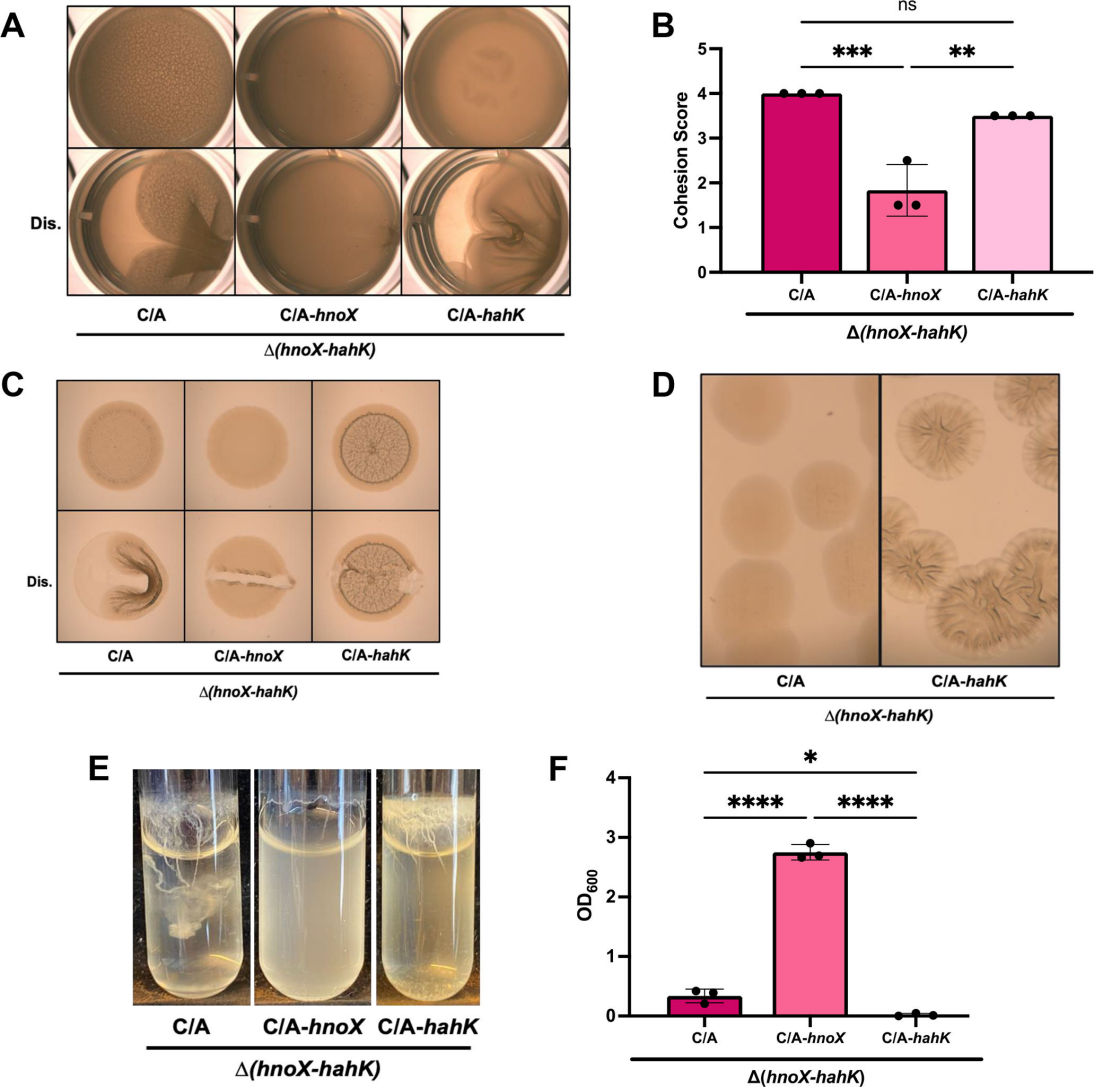
An *hnoX* promoter mutation increases pellicle formation dependent on *hahK*. For all panels except D, the same three strains were tested. The strains were built using the Δ(*hnoX-hahK*) parent strain and contained, at a non-native locus, P*hnoX-*C/A-*hnoX-hahK* (C/A; KV10620), P*hnoX*-C/A-*hnoX* (C/A-*hnoX*; KV10711), or P*hnoX-*C/A-*hahK* (C/A-*hahK*; KV10770). (**A**) Representative images of pellicles grown statically for 72 h at 24°C in LBS + 10 mM Ca^2+^. Top, undisturbed; bottom, disturbed with a toothpick (Dis.). (**B**) Biofilm cohesion score for pellicles similar to and including those shown in panel A. **, *P* **≤** 0.01; ***, *P* **≤** 0.001; ns, not significant. Error bars represent SD. (**C**) Representative images of biofilms formed on solid LBS agar media containing 10 mM Ca^2+^ and incubated for 72 h at 24°C. Top, undisturbed; bottom, disturbed with a toothpick (Dis.). (**D**) Representative images of single colonies of each strain streaked onto solid LBS agar media incubated at 28°C for ~40 h. Both strains were built using the ∆(*hnoX-hahK*) parent strain and contained P*hnoX*-C/A-*hnoX-hahK* (C/A; KV10620) or P*hnoX*-C/A-*hahK* (C/A-*hahK*; KV10770). (**E**) Representative images of biofilms formed under shaking conditions by cells grown for 24 h at 24°C in tTBS with 10 mM Ca^2+^. (**F**) OD_600_ measurements of the liquid surrounding the biofilms similar to and including those shown in panel E. *, *P* ≤ 0.05; ****, *P* < 0.0001. Error bars represent SD.

Past work revealed HnoX and HahK to be negative and positive regulators, respectively, of biofilm formation (11, 21). The C/A point mutation presumably drives transcription of both genes, but because HahK promotes biofilm formation, we hypothesized that the pellicle phenotype of strains carrying the C/A point mutation was, ultimately, due to an increase in levels of HahK. To test the requirement for the two regulators, we evaluated the biofilm competence of strains that carried the C/A change upstream of either *hnoX* or *hahK* alone in comparison to the C/A-*hnoX-hahK* (C/A-operon; “C/A”)-carrying strain. The C/A change upstream of *hahK*, but not *hnoX*, significantly promoted cohesive pellicle formation (Fig. 6A and B). We note that pellicles produced by the C/A-*hahK* strain appeared visually different from the pellicles produced by the C/A-operon-carrying strain by the generation of large folds upon disruption of the pellicle; this contrasts with the smaller architectural wrinkles observed prior to disruption for the C/A-operon-carrying strain (Fig. 6A). To explore these differences further, we examined pellicle formation at an earlier timepoint (24 h instead of 72 h). At 24 h, the C/A-*hahK* strain produced pellicles with clear cohesion not seen for control strains, including the C/A-operon strain (Fig. S4), suggesting that the former strain produced cohesive biofilms earlier. Together, these data support the conclusions that (i) the C/A change impacts biofilm formation by increasing the production of HahK, and (ii) HnoX exerts some control over HahK under these conditions. However, given that the C/A-operon strain also forms enhanced pellicles, we conclude that the control by HnoX is not sufficient to prevent all induction of biofilm formation by HahK.

Consistent with the cohesive pellicles produced by the C/A-*hahK* strain, this strain produced robustly wrinkled spots on LBS plates that contained calcium; indeed, they attached to the agar medium, a phenomenon that did not occur for the C/A-operon strain (Fig. 6C). Furthermore, the two strains differed in their properties when streaked onto LBS plates that lacked added calcium: the C/A-*hahK* strain formed small colonies with a wrinkled colony architecture evident within 48 h, while the C/A-operon strain formed WT-like colonies (Fig. 6D). In contrast, the C/A-operon strain formed visually better biofilms under shaking conditions, although culture clarity was similar (Fig. 6E and F). In both of these assays, as with pellicle formation, the C/A-*hnoX* strain did not produce robust biofilms (Fig. 6C, E and F). Together, these data indicate that the activity of HnoX under the tested conditions is sufficient to diminish but not completely prevent HahK activity. Because HnoX is known to bind NO (17), it seems likely that insufficient NO levels exist under these laboratory conditions to fully control HahK activity.

### Biofilms of the *hnoX* promoter mutant depend fully on *sypG*, but only partially on *sypF*

**Fig 7:**
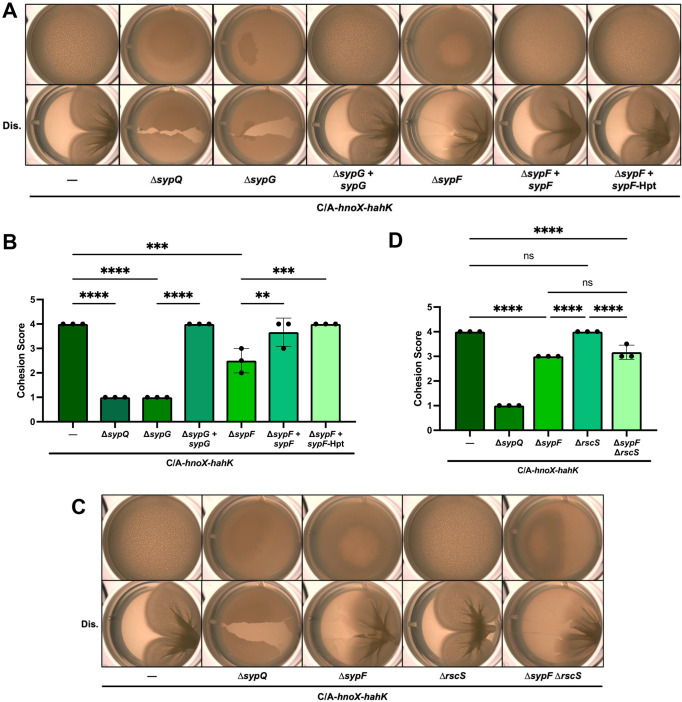
*hnoX* promoter mutant biofilms depend fully on *sypG*, but only partially on *sypF*. (**A**) Representative images of pellicles produced by strains that carry the P*hnoX*-C/A-*hnoX-hahK* operon (C/A-*hnoX-hahK*) at a non-native locus in otherwise WT background (—; KV10701) and containing *syp* mutations as follows: Δ*sypQ* (KV10700), Δ*sypG* (KV10652), Δ*sypG* complemented with *sypG* (Δ*sypG + sypG*; KV10663), Δ*sypF* (KV10650), Δ*sypF* complemented with *sypF* (Δ*sypF + sypF*; KV10661), and Δ*sypF* complemented only with the Hpt domain of *sypF* (Δ*sypF + sypF* Hpt; KV10662). Cells were grown in LBS with 10 mM Ca^2+^ for 72 h at 24°C. Top, undisturbed; bottom, disturbed with a toothpick (Dis.). (**B**) Biofilm cohesion scores for pellicles similar to and including those in panel A. **, *P* **≤** 0.01; ***, *P* **≤** 0.001; ****, *P* < 0.0001. Error bars represent SD. (**C**) Representative images of pellicles produced by strains that carry the P*hnoX-*C/A-*hnoX-hahK* operon (C/A-*hnoX-hahK*) at a non-native locus in otherwise WT background (—; KV10701) and containing *syp* or *rscS* mutations as follows: Δ*sypQ* (KV10700), Δ*sypF* (KV10650), Δ*rscS* (KV10651), and Δ*sypF* Δ*rscS* (KV10837). Cells were grown in LBS with 10 mM Ca^2+^ for 72 h at 24°C. Top, undisturbed; bottom, disturbed with a toothpick (Dis.). (**D**) Biofilm cohesion scores for pellicles similar to and including those in panel C. ****, *P* < 0.0001; ns, not significant. Error bars represent SD.

HahK, a histidine kinase, was previously reported to activate *syp*-dependent biofilm formation via the two-component pair SypF and SypG (11). Thus, to understand the consequences on biofilm formation of the upregulation of HahK caused by the C/A change, we tested the dependence of the biofilm phenotypes on the *syp* locus and its regulators. Deletion of the structural *syp* gene *sypQ* or the gene for the proximal DNA-binding regulator SypG eliminated cohesive pellicle formation; complementation of Δ*sypG* with *sypG* restored biofilm formation (Fig. 7A and B). These data support the conclusion that the C/A change upstream of the *hnoX-hahK* operon induces *syp*-dependent biofilms.

When we evaluated a strain that carried the C/A*-*operon insertion but lacked the upstream sensor kinase SypF, we observed the production of sticky pellicles; although architecture was diminished and overall stickiness reduced, pellicle production was not eliminated (Fig. 7A and B). This result was surprising as several previous studies had demonstrated the requirement for the Hpt domain of SypF for biofilm formation (11, 49, 50). We confirmed this result both by remaking the original Δ*sypF* C/A-*hnoX-hahK* strain and by using a different Δ*sypF* background (Fig. S5), experiments that yielded the same outcomes observed in Fig. 7. Further demonstrating the validity of the diminished biofilm phenotype, the Δ*sypF* mutant could be complemented to full biofilm formation by expression of either the Hpt domain or full-length SypF (Fig. 7A and B).

In addition to control via HahK, SypF is also activated under some conditions by the sensor kinase RscS (8, 13, 49) (Fig. 1A). Deletion of *rscS* did not impact cohesive pellicle formation induced by the *hnoX* promoter mutation, either alone or when paired with a Δ*sypF* deletion (Fig. 7C and D); the double Δ*rscS* Δ*sypF* mutant phenocopied the Δ*sypF* mutant. Thus, the partial phenotype of the Δ*sypF* mutant indicates that HahK must work through an additional regulator(s) besides SypF to control *syp*-dependent biofilm formation.

### LuxU contributes to biofilms formed by HahK overexpression

**Fig 8:**
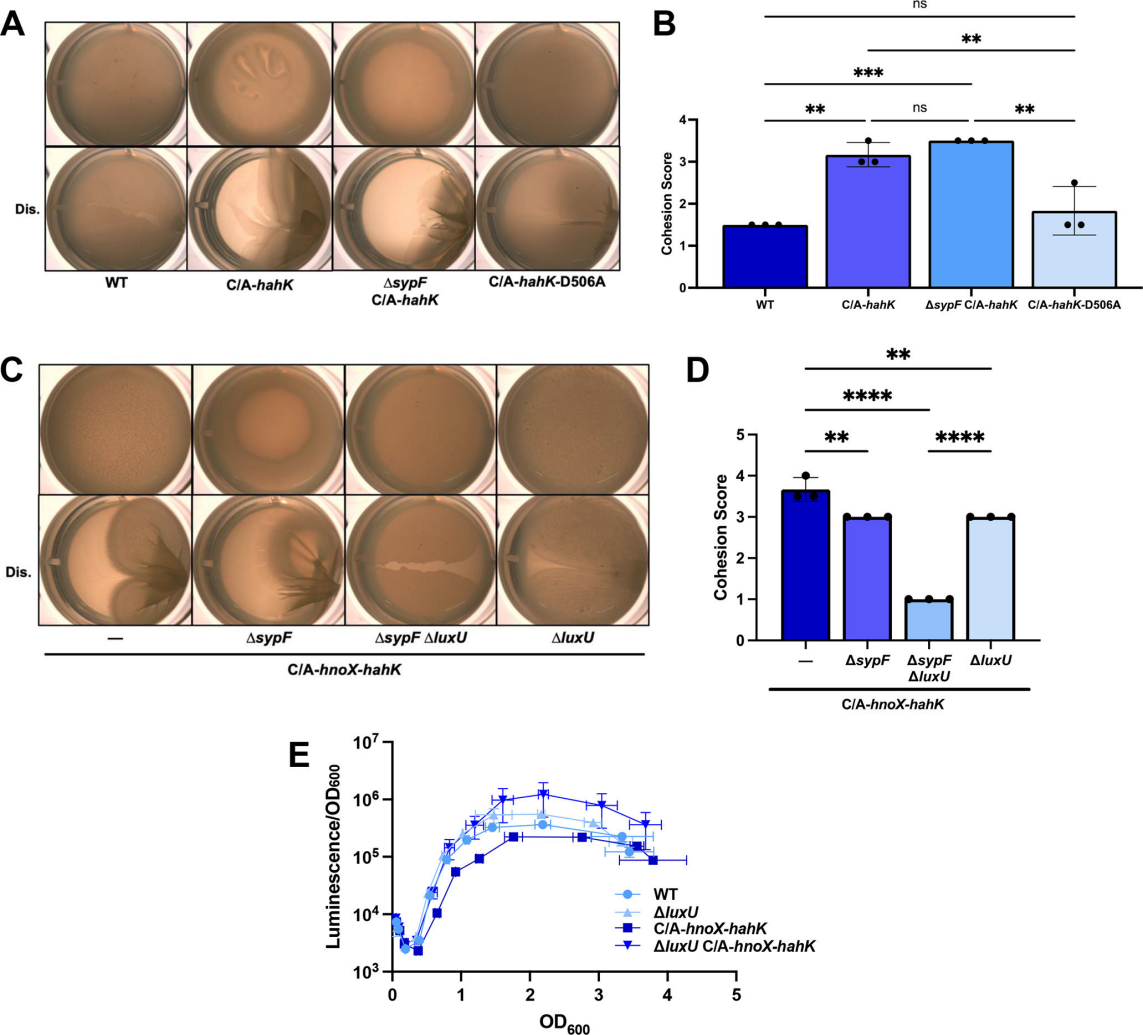
Impact of *syp* regulatory mutations on HahK-induced biofilm formation. (**A**) Representative pellicle images of ES114 (WT) and strains containing P*hnoX*-C/A-*hahK* (C/A-*hahK*; KV10838), P*hnoX-*C/A-*hahK* Δ*sypF* (∆*sypF* C/A-*hahK*; KV10829), and P*hnoX*-C/A-*hahK-*D506A (C/A-*hahK*-D506A; KV10933). Cells were grown statically for 72 h at 24°C in LBS + 10 mM Ca^2+^. Top, undisturbed; bottom, disturbed with a toothpick (Dis.). (**B**) Biofilm cohesion score for pellicles similar to and including those in panel A. **, *P* ≤ 0.01; ***, *P* ≤ 0.001; ns, not significant. Error bars represent SD. (**C**) Representative images of pellicles produced by strains that carry a copy of the P*hnoX*-C/A-*hnoX-hahK* operon (C/A-*hnoX-hahK*) at a non-native locus in otherwise WT (—; KV10701), Δ*sypF* (KV10650), Δ*sypF* Δ*luxU* (KV10962), and Δ*luxU* (KV10963) backgrounds. Cells were grown statically at 24°C for 72 h in LBS + 10 mM Ca^2+^. Top, undisturbed; bottom, disturbed with a toothpick (Dis.). (**D**) Biofilm cohesion score for pellicles similar to and including those in panel C. **, *P* ≤ 0.01; ****, *P* < 0.0001. Error bars represent SD. (**E**) Specific luminescence (relative light units divided by OD_600_) plotted against OD_600_ of ES114 (WT), Δ*luxU* (KV10846), a P*hnoX*-C/A-*hnoX-hahK* containing strain (C/A-*hnoX-hahK*; KV10701), and a P*hnoX*-C/A-*hnoX-hahK* containing Δ*luxU* mutant (Δ*luxU* C/A-*hnoX-hahK*; KV10963). Cells were grown with shaking in SWTO at 24°C. Error bars represent SD along both the X-axis and Y-axis. Some error bars are not visible because they are smaller than the size of the symbol.

Because disruption of *sypF*, either alone or in combination with deletion of *rscS*, failed to prevent pellicle production by strains carrying the C/A mutation, we explored alternative explanations for pellicle production. First, we hypothesized that, under these conditions, HahK could donate its phosphoryl group from the conserved site of phosphorylation, H222, directly to SypG; H222 has previously been shown to be critical for biofilm formation (51). If phosphorylation could proceed from H222 to SypG, then the conserved aspartate, D506, in the receiver domain of HahK, which is also critical for biofilm formation (51), would be dispensable. Thus, we evaluated biofilm formation by a C/A-*hahK*-D506A strain and corresponding controls. As seen previously in the context of the full operon (Fig. 7), the loss of SypF diminished but did not fully disrupt biofilm formation by the C/A-*hahK*-expressing strain (Fig. 8A and B). When expressed in an otherwise WT background under the control of P*hnoX*-C/A, HahK-D506A failed to promote the production of strong pellicles like its WT parent; instead, this strain largely phenocopied WT strain ES114 (Fig. 8A and B). These data suggest that it is unlikely that HahK promotes biofilm formation by directly signaling to SypG.

Given the requirement for the D506 residue of HahK, we considered whether HahK could rely on an Hpt domain other than that of SypF. Previous work has shown that LuxU, a phosphotransferase in the luminescence (*lux*) pathway, could influence SypG activity when the latter protein was expressed from a multi-copy plasmid (22). Indeed, the loss of LuxU disrupted the residual biofilm formation of the Δ*sypF* mutant that expresses the C/A-*hnoX-hahK* operon (Fig. 8C and D). Furthermore, the loss of LuxU alone modestly diminished cohesive pellicle formation by the C/A-*hnoX-hahK* strain (Fig. 8C and D).

Because (i) LuxU was required for *hahK*-induced biofilm formation and (ii) phosphorylated LuxU inhibits bioluminescence in *V. fischeri* (25, 26, 52), we asked if strains that carry the C/A change upstream of the *hnoX-hahK* operon produced lower levels of bioluminescence. Indeed, the C/A-operon-containing strain produced levels of light that consistently trended lower relative to the WT strain (Fig. 8E). This defect was overcome by disruption of *luxU* (Fig. 8E). These results are consistent with previous work in *Vibrio harveyi* that determined that HnoX and its corresponding sensor kinase HqsK controls luminescence via LuxU (53). Together, these data indicate that HahK can signal through both SypF and LuxU to influence SypG-dependent biofilm formation as well as through LuxU to influence luminescence.

### *hahK* overexpression promotes colonization in the absence of SypF

**Fig 9:**
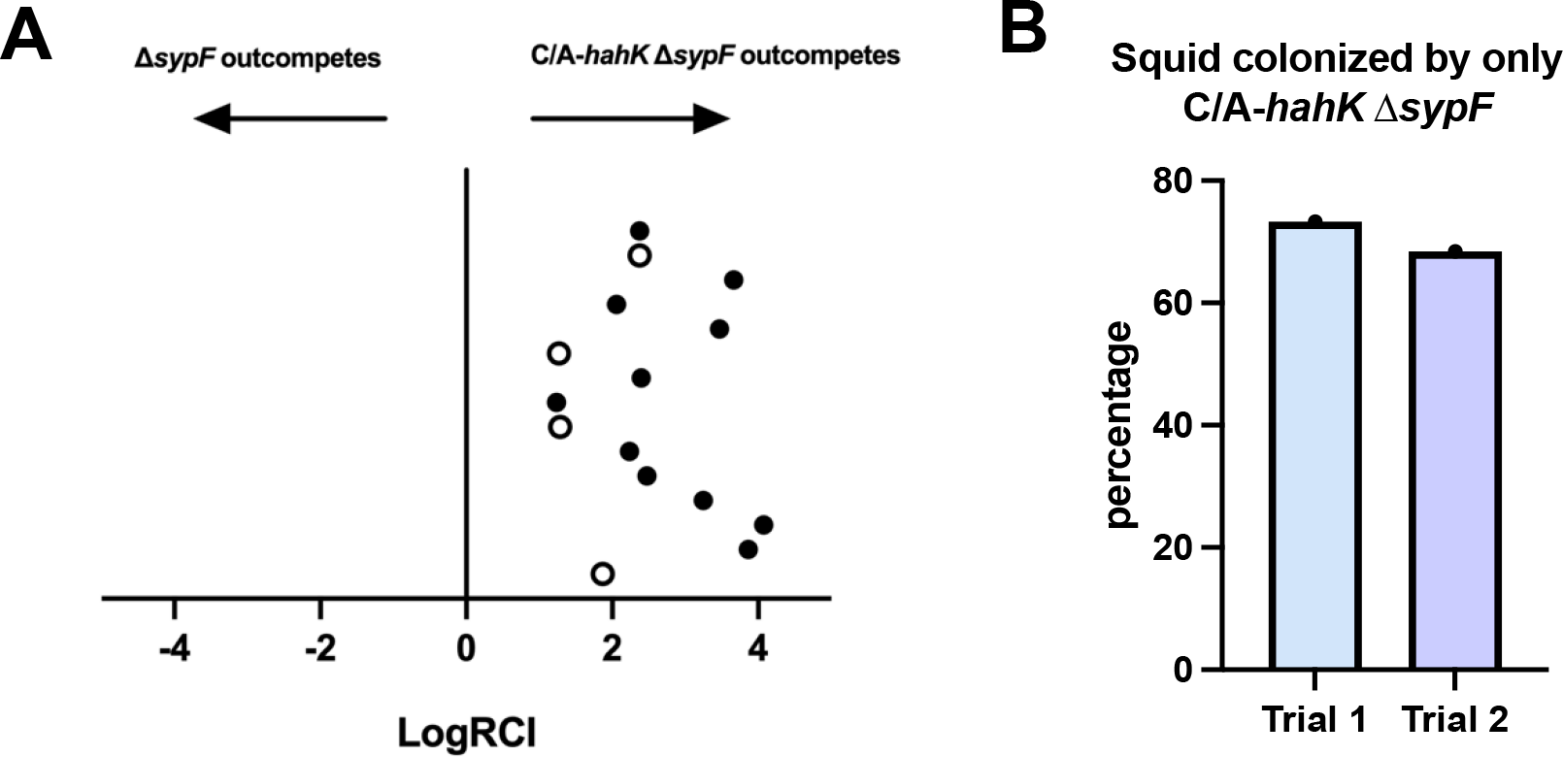
*hahK* overexpression promotes colonization of juvenile squid in the absence of SypF. (**A**) Newly hatched *E. scolopes* juveniles were exposed to two competing strains, one containing the P*hnoX*-C/A*-hahK* allele (C/A-*hahK*) in a Δ*sypF* mutant background (KV10822) and the other only containing the Δ*sypF* mutation (KV9931). Juveniles were colonized for ~24 h before euthanizing, homogenizing, and plating to calculate CFU/squid. The LogRCI was calculated by dividing the ratio of C/A*-hahK* Δ*sypF*:Δ*sypF* in the animal by the same ratio in the initial inoculum; a LogRCI > 0 means that strain KV10822 dominated in the competition. Closed circles represent squid that contained KV9931 below the limit of detection. Sample size = 15, repeated once with similar results. (**B**) For the experiment shown in panel A (trial 1) and a second independent experiment (trial 2), the percentage of juvenile squid that were colonized exclusively by C/A*-hahK* Δ*sypF* strain KV10822 was plotted.

Because biofilms could form in the absence of *sypF* when *hahK* was overexpressed, we wondered if overexpression of *hahK* would result in a colonization advantage. We thus performed a colonization competition by exposing newly hatched juvenile squid to a Δ*sypF* mutant and a Δ*sypF* mutant that carried the C/A-*hahK* allele. Within 24 h, the latter strain dominated in the juvenile squid (Fig. 9A). Indeed, in each of the two experiments, over 60% of animals were exclusively colonized by the Δ*sypF* mutant carrying the C/A-*hahK* allele (Fig. 9B), indicating that colonization defects stemming from the loss of SypF can be suppressed by *hahK* overexpression. Overall, these data confirm that HahK can function through a SypF-independent pathway to promote host colonization, presumably via LuxU.

### The *hnoX* promoter region differs between *V. fischeri* isolates

**Fig 10:**
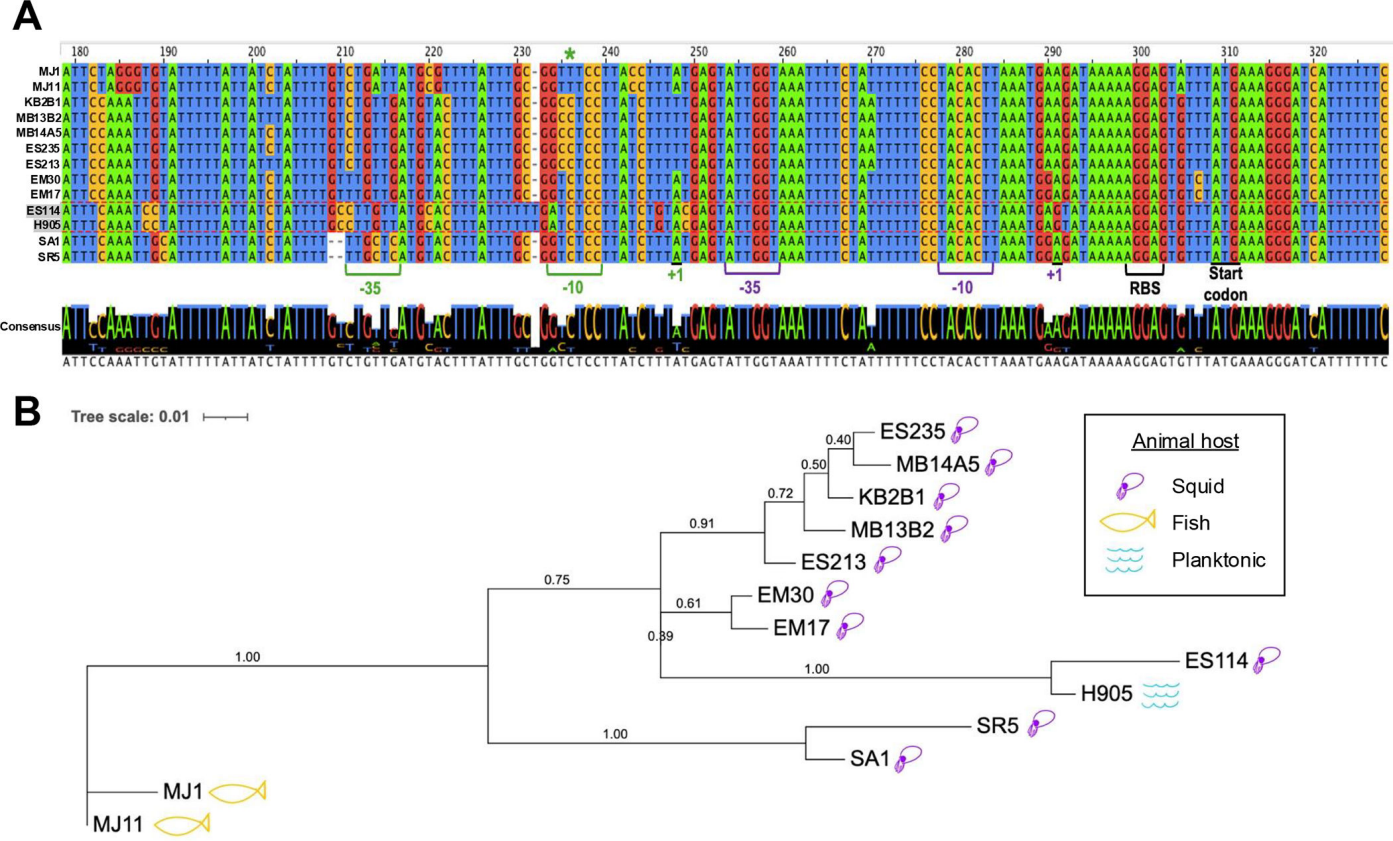
Alignment of *hnoX* promoter sequences. (**A**) Top, alignment of sequences for the indicated *V. fischeri* strains across ~144 bases from just upstream of the BPROM-predicted −35 sequence to 13 bases past the *hnoX* start codon. The specific isolates include MJ1, MJ11, KB2B1, MB13B2, MB14A5, ES235, ES213, EM30, EM17, ES114, H905, SA1, and SR5. The transcriptional start sites (+1) and predicted promoters (−35 and −10) for WT ES114 and for the C/A mutant are shown in purple and green text, respectively. The critical C base is indicated at the top with a green asterisk. The ribosome-binding site (RBS) and start codon are also indicated. Bottom, consensus sequence. (**B**) Phylogenetic relationships of the strains based on the *hnoX* promoter sequences aligned in panel A. The scale bar indicates an evolutionary distance of 0.01 nucleotide substitutions per position in the sequence. The numbers on the nodes represent the bootstrap statistical support as fractional values.

Although we used ES114 to assess the impact of the C/A point mutation on *V. fischeri*, other squid- and fish-colonizing isolates have been characterized. To determine the conservation of the critical C base of the *hnoX* promoter, this region was PCR amplified from the chromosomal DNA of a collection of *V. fischeri* isolates, and the products were sequenced. We also obtained the sequence of one non-symbiotic isolate, H905, from Genbank (54). The resulting alignment of the relevant region is shown in Fig. 10A. The critical C base (green asterisk) in the *hnoX* regulatory region is conserved in all isolates from *E. scolopes* but is replaced with a T in the two fish isolates, MJ1 and MJ11. The sequences of all examined strains have identical predicted −10 and −35 sites (purple brackets) upstream of the transcription start site (purple +1) experimentally determined by 5' RACE for WT strain ES114. From the −35 motif and downstream sequences leading into the coding region for *hnoX*, the alignment displays mostly identical bases. In the region where the C/A change generates an alternative promoter (−10 and −35 sequences bracketed in green), however, there is a notable divergence of nucleotide sequences. In the other isolates, the sequence aligned to the alternate −10 sequence contains a G instead of the A found in ES114 and H905, and a GC nucleotide couplet exists immediately upstream in place of the TT present in ES114 and H905. Similarly, in the −35 region of the promoter generated by the C/A change, there are differences in the sequence where the T nucleotides found in ES114 and H905 (TT*G*TTA) are replaced by G nucleotides in the other squid isolates (TG*T*TGA). The alignment of the *hnoX* promoter sequences alone results in a phylogenetic tree that resembles that produced by the whole-genome comparison of some of the same strains in a previous study (Fig. 10B) (55) in regard to topology and grouping of the strains. The ES114 strain is positioned similarly in both trees on a branch separate from other *E. scolopes* strains. These data indicate that, in ES114, the sequence involved in the regulation of the *hnoX/hahK* operon has diverged from other *E. scolopes* isolates in a manner analogous to other regions of the genome that distinguish the isolates.

## DISCUSSION

SYP is a major contributor to *V. fischeri* biofilms and host colonization (3, 5). *V. fischeri* exerts considerable resources to control both *syp* transcription and SYP production, including input from multiple sensor kinases and at least two response regulators (4, 6, 8, 10, 11, 13, 14, 22, 56). Past work had identified HahK as a regulator of SYP-dependent phenotypes (11), although its contribution in culture is masked by the activities of other regulators. Here, we identified a single C/A point mutation upstream of the *hnoX-hahK* operon that resulted in robust SYP-dependent biofilm formation caused by a significant increase in *hahK* transcription. Single nucleotide changes have previously been shown to alter codons and thus protein function and/or to generate new promoters, resulting in altered gene transcription (57, 58) and leading to new insights in regulation. By studying the consequences of the C/A mutation, we expanded our understanding of the regulatory pathway for SYP production through the identification of Zur as an inhibitor of *hnoX-hahK* transcription and of the luminescence regulator LuxU as a downstream regulator for HahK-induced cohesive biofilm formation in *V. fischeri*.

The C/A change upstream of *hnoX-hahK* arose during an enrichment for pellicle production by a strain carrying two copies of the gene for the transcription factor LitR. While well-studied for its role in promoting luminescence, LitR has only recently been shown to inhibit biofilm formation in *V. fischeri* (Fig. 1A), in part by controlling the production of the polysaccharide cellulose (27–29, 31). Thus, our initial goal was to identify additional factors that LitR might control that inhibit biofilm formation. However, neither LitR nor its upstream regulator Qrr1 influenced *hnoX-hahK* transcription, suggesting that the C/A change represented a bypass suppressor mutation. While this may be the case, our finding that HahK-induced biofilm formation was partially dependent on LuxU presents the possibility that HahK could also promote biofilm formation by decreasing the levels of the inhibitory LitR protein. LuxU thus appears to be another pivotal branch point in the pathway, with its ability to influence both luminescence nd biofilm formation via the LuxO-Qrr1-LitR arm (26, 29, 59) and biofilm formation via SypG, as previously implicated (22). Our data connecting HahK to LuxU provide another control mechanism and build on the growing body of work establishing interconnectivity between the bioluminescence and biofilm pathways, work that also includes the activation of *qrr1* transcription by SypG and the putative oligosaccharide translocase SypK (23, 24). It is evident that more research is needed to fully explore the links between these important *V. fischeri* processes.

Our initial efforts to understand the impact of the C/A change relied on a promoter prediction garnered through the use of BPROM (32). This is a useful program, but in this case, it failed to accurately predict the *hnoX* promoter. *V. fischeri* has a low G + C content (38.3%) (18), making it difficult to predict promoters. Our results should serve as a note of caution for *V. fischeri* researchers—and potentially others who study low G + C content bacteria. As more data are obtained from RNAseq and other large-scale analyses, there will likely be less need to rely on such prediction software.

It is expected that some of the impact of HahK overproduction on SYP-dependent biofilm formation occurs at the level of *syp* transcription via SypG. However, control of SYP production does not occur exclusively at the level of *syp* transcription. Instead, the response regulator SypE functions at a level below *syp* transcription by controlling the activity of SypA, which is required for biofilm formation (6, 13). The phosphorylation state of SypE, and thus activity, is thought to be controlled by SypF (50); phosphorylated SypE favors biofilm formation, while unphosphorylated SypE inhibits it (6, 10, 56). However, strains with the C/A change did not need SypF for pellicle formation (Fig. 7). If SypF is the only regulator that controls SypE phosphorylation, then, in the absence of SypF, SypE should be unphosphorylated and thus biofilm inhibitory. Given that the C/A-operon-containing Δ*sypF* mutant can produce a reasonably robust pellicle that is dependent on LuxU, we speculate that either (i) the increased HahK-dependent activation of LuxU, potentially causing phosphorylation of SypG and thus increased *syp* transcription, is sufficient to overcome inhibition by SypE, or (ii) in this genetic background, LuxU can also phosphorylate and inactivate SypE. We favor the latter possibility, as overproduction of SypG from a multi-copy plasmid is insufficient to produce robust *syp*-dependent biofilms if *sypE* is intact (13). Understanding how LuxU influences *syp*-dependent biofilm formation is another important area of future research.

In the squid host, NO is produced in vesicles within the mucus shed by ciliated epithelial cells on the surface of the light organ (60). The initial concentration of NO is presumably low enough such that *V. fischeri* can successfully form HahK-dependent symbiotic aggregates, yet high enough to drive the specificity of the symbiotic relationship (21, 61). Potentially, the NO levels may subsequently increase, leading to HnoX-mediated inhibition of HahK kinase activity and diminished biofilm formation. Whether HahK functions as a phosphatase remains unknown, but if it does, such phosphatase activity could contribute to facilitating dispersal—perhaps via both the SypF-SypG arm and LuxU-mediated processes.

In the context of the C/A change upstream of the full operon, HnoX did not prevent HahK activity *in vitro*, suggesting that the levels of NO, if any, in our laboratory conditions are not sufficient for full HnoX-mediated inhibition. In our squid assays, we used a strain with the C/A change upstream of *hahK* that also lacked SypF. This strain could robustly outcompete the Δ*sypF* mutant, suggesting that it could promote *syp*-dependent symbiotic aggregates, presumably through LuxU, to facilitate colonization. These data thus support the relevance of the *in vitro* studies. We note that the strain in the squid colonization experiments retained its native copy of the *hnoX-hahK* operon, suggesting that HnoX may have contributed to control over HahK in the context of the squid experiment. In any event, we anticipate that strains developed in this study have the potential to be used to understand the levels of NO present under different conditions and the importance of inactivating HahK in the context of symbiosis. For example, we would expect that high levels of NO would lead to decreased biofilm formation by the strain carrying the C/A point mutation. Additionally, it would be of interest to determine, in future work, if strains that carry the C/A change upstream of the operon would have a colonization advantage over the WT strain or if the *in vivo* levels of NO sufficiently modulate HahK activity such that WT-like behavior occurs.

The observation that the *hnoX* regulatory region shows a divergence in sequence between ES114 and other *V. fischeri* isolates is intriguing. Specifically, the changes seem to either increase the G + C content of the region surrounding the critical C base or replace/flip the T bases with G or C bases upstream in the region corresponding to the “new” −35. Given the low G + C content of *V. fischeri* (18), an increase in the local intergenic G + C content may be responsible for some differences in strain behavior. In the genomic comparison of various strains that were classified as “dominant” (isolates that outcompete others for squid colonization) or “sharing” (isolates that productively co-colonize), ES114 was positioned in the latter class (55). In that study, no individual proteins were identified as specific to dominant strains only. It was hypothesized that differential strain behavior may be due to allelic differences in shared genes (55, 62, 63). Here, we identified allelic differences in the non-coding regulatory region for a conserved operon, *hnoX-hahK*, that may have allowed more rapid evolution of ES114 to increase SYP production. Indeed, it seems unlikely that the C/A change alone, if generated in the other isolates analyzed here (with the possible exception of H905), would result in the increased biofilm phenotypes observed for ES114. Additional study of multiple *V. fischeri* isolates will undoubtedly drive forward our understanding of the evolutionary changes that lead to productive host colonization.

## MATERIALS AND METHODS

### Strains, growth media, and conditions

*V. fischeri* strain ES114 (64) was used as the parent strain for these studies. Derivatives of ES114 were constructed as described below, and the final strains used are listed in Table 1. *V. fischeri* was routinely cultured at 28°C in Luria-Bertani salts (LBS) medium (65) (1% tryptone, 0.5% yeast extract, 2% sodium chloride, and 50 mM Tris pH 7.5), amended as appropriate with antibiotics as listed below. Experiments made use of both LBS and tris-buffered tryptone broth salt (tTBS) (1% tryptone, 2% sodium chloride, and 50 mM Tris pH 7.5) (9), containing or lacking 10 mM calcium chloride; cells were grown at 28°C or 24°C as indicated. In addition, Seawater Tryptone (SWT) (0.5% tryptone, 0.3% yeast extract, 35 mM MgSO_4_, 7 mM each KCl and CaCl_2_, and 210 mM NaCl) was used for squid experiments (65); SWT was amended with 20 g/L NaCl to make SWTO (66), which was used for luminescence experiments. Tris minimal medium was used for transformations and contained 100 mM Tris pH 7.5, 300 mM NaCl, 50 mM MgSO_4_, 0.33 mM K_2_HPO_4_, 10 µM ferrous ammonium sulfate, 0.1% NH_4_Cl, 10 mM N-acetylglucosamine, and 10 mM KCl (67). In some experiments, ZnCl_2_ was added to final concentrations ranging from 0.5 to 2 mM as noted. The final concentrations of antibiotics used for selection in *V. fischeri* were as follows: chloramphenicol (Cm; 1 µg/mL), erythromycin (Erm; 2.5 µg/mL), kanamycin (Kan; 100 µg/mL), spectinomycin (Spec; 40 µg/mL).

**TABLE 1:** *V. fischeri* strains used in this study

Name	Genotype^*a*^	Reference
EM17	WT isolate (squid)	68, 69
EM30	WT isolate (squid)	68
ES114	WT isolate (squid)	64
ES213	WT isolate (squid)	70
ES235	WT isolate (squid)	70
KB2B1	WT isolate (squid)	71
MB13B2	WT isolate (squid)	71
MB14A5	WT isolate (squid)	71
MJ1	WT isolate (fish)	72
MJ11	WT isolate (fish)	68, 73
SA1	WT isolate (squid)	74
SR5	WT isolate (squid)	74
BF450	Δ*sypQ*::FRT IG::P*hnoX*-lacZ	This study
BF451	Δ*sypQ*::FRT IG::P*hnoX-lacZ* Δ*litR*::FRT	This study
BF478	Δ*sypQ*::FRT IG::P*hnoX* (C/A)-*lacZ*	This study
KV9931	Δ*sypF*::FRT	This study
KV10050	IG (Erm^R^)::P*litR-litR*	29
KV10511	Δ(*hnoX-hahK*)::FRT	This study
KV10513	Δ(*hnoX-hahK*)::FRT IG::P*hnoX-hnoX-hahK*-HA	This study
KV10528	*zur*::Tn-Mariner	This study
KV10552	Δ*zur*::FRT-Erm^R^	This study
KV10564	Δ*zur*::FRT	This study
KV10620	Δ(*hnoX-hahK*)::FRT IG::P*hnoX* (C/A)-*hnoX-hahK*-HA	This study
KV10645	Δ*sypQ*::FRT IG::P*hnoX* (C/G)-*lacZ*	This study
KV10646	Δ*sypQ*::FRT IG::P*hnoX* (C/T)-*lacZ*	This study
KV10648	Δ*sypQ*::FRT IG::P*hnoX*-Trunc-1-*lacZ*	This study
KV10649	ΔsypQ::FRT IG::P*hnoX*-Trunc-2-*lacZ*	This study
KV10650	Δ*sypF* IG::P*hnoX* (C/A)-*hnoX-hahK*-HA	This study
KV10651	Δ*rscS* IG::P*hnoX* (C/A)-*hnoX-hahK*-HA	This study
KV10652	Δ*sypG* IG::P*hnoX* (C/A)-*hnoX-hahK*-HA	This study
KV10653	IG::P*hnoX-hnoX-hahK*-HA	This study
KV10661	Δ*sypF* IG::P*hnoX* (C/A)-*hnoX-hahK*-HA attTn*7*::*sypF*-flag	This study
KV10662	Δ*sypF* IG::P*hnoX* (C/A)-*hnoX-hahK*-HA attTn*7*::*sypF*-Hpt-flag	This study
KV10663	Δ*sypG* IG::P*hnoX* (C/A)-*hnoX-hahK*-HA attTn*7*::*sypG*-flag	This study
KV10666	Δ*sypQ*::FRT IG::P*hnoX-lacZ* Δ*qrr1*::FRT-Trim^R^	This study
KV10668	Δ*sypQ*::FRT IG::P*hnoX-lacZ* Δ*zur*::FRT-Erm^R^	This study
KV10696	Δ*sypQ*::FRT IG::P*hnoX-lacZ zur*::Tn-Mariner	This study
KV10700	Δ*sypQ*::FRT IG::P*hnoX* (C/A)-*hnoX-hahK*-HA	This study
KV10701	IG::P*hnoX* (C/A)-*hnoX-hahK*-HA	This study
KV10708	IG (Erm^R^)::P*litR-litR* + C74297A (and other possible changes)	This study
KV10711	Δ(*hnoX-hahK*)::FRT IG::P*hnoX* (C/A)-*hnoX*-HA	This study
KV10770	Δ(*hnoX-hahK*)::FRT IG::P*hnoX* (C/A)-*hahK*-HA	This study
KV10772	Δ*sypQ*::FRT IG::P*hnoX*-Trunc-3-*lacZ*	This study
KV10822	Δ*sypF*::FRT IG (Erm^R^)::P*hnoX* (C/A)-*hnoX-hahK*-HA	This study
KV10829	Δ*sypF* IG::P*hnoX* (C/A)-*hahK*-HA	This study
KV10833	Δ*sypF*::FRT IG::P*hnoX* (C/A)-*hnoX-hahK*-HA	This study
KV10837	Δ*rscS* Δ*sypF* IG::P*hnoX* (C/A)-*hnoX-hahK*-HA	This study
KV10838	IG::P*hnoX* (C/A)-*hahK*-HA	This study
KV10841	Δ*sypF* IG::P*hnoX* (C/A)-*hnoX-hahK*-HA	This study
KV10846	Δ*luxU*::FRT-Spec^R^	This study
KV10933	IG::P*hnoX* (C/A)-*hahK*-D506A-HA	This study
KV10962	Δ*sypF* IG::P*hnoX* (C/A)-*hnoX-hahK*-HA Δ*luxU*::FRT-Spec^R^	This study
KV10963	IG::P*hnoX* (C/A)-*hnoX-hahK*-HA Δ*luxU*::FRT-Spec^R^	This study

^*a*^ Genotype abbreviations are as follows: intergenic region (IG) between the genes *yeiR* and *glmS*, generated using FRT-flanked Erm^R^; IG (Erm^R^; shorthand for IG [*yeiR*-FRT-Erm^R^/*glmS*]); derivative in which the Erm^R^ cassette has been removed, IG (shorthand for IG [*yeiR*-FRT/*glmS*]); attTn*7*, insertion of Tn*7* at the Tn*7 att* site between *yeiR* and *glmS*; C/A, C74297A, the point mutation upstream of the *hnoX* promoter, and correspondingly, C/T and C/G changes; HA, hemagglutinin epitope tag; flag, flag epitope tag; D506A, aspartate to glutamate substitution at amino acid 506; Trunc, truncation; animals within parentheses following WT isolates indicate the host animal from which the strain was isolated.

*Escherichia coli* strains were used for the purposes of plasmid maintenance and conjugation as described previously (67, 75) and included π3813 (76), CC118λ*pir* (77), Tam1 λ *pir* (Active Motif), GT115 (Invivogen), S17-1 λ*pir* (78), and β3914 (76). *E. coli* strains were grown in lysogeny broth (LB) medium amended as appropriate with thymidine (0.3 mM), diaminopimelic acid (DAP) (0.3 mM), and/or with antibiotics as follows: Cm (12.5 µg/mL) and Kan (50 µg/mL).

### Strain construction

To obtain the final strains used in these studies (Table 1), strain ES114 and its derivatives were genetically manipulated as described in Table S1 using plasmids listed in Table S2 and primers listed in Table S3. Strains engineered to carry insertions or deletions were generated using PCR and Splicing by Overlap Extension (67), and the resulting DNA products were transformed into *tfoX*-overexpressing strains as previously described (67, 75, 79). Tri-parental conjugations were used to introduce *tfoX* overexpression plasmids or flippase plasmid pKV496 into specific *V. fischeri* strains to promote uptake of exogenous DNA or resolve flippase recombination target (FRT) -flanked antibiotic resistance cassettes as previously described (67, 75, 79, 80). For complementation using Tn*7*, tetra-parental matings were used as previously described (67), which included the *V. fischeri* recipient, an *E. coli* donor that carried the Tn*7*-containing plasmid, an *E. coli* helper strain that carried pEVS104 (81), and an *E. coli* strain that carried the Tn*7* transposase plasmid pUX-BF13 (82).

### Pellicle enrichment

*V. fischeri* strains were grown overnight in LBS at 24°C with shaking. The next morning, strains were subcultured into 24-well plates at an OD_600_ of 0.02 in 2 mL of LBS-Ca. The plates were incubated at 24°C for 72 h in static conditions. Following this incubation, the pellicles (or any cells at the surface of the liquid) were reinoculated (i) directly into new 24-well plates in 2 mL of LBS-Ca and incubated for 72 h at 24°C again or (ii) into 5 mL LBS to proceed with the overnight to repeat the process from the beginning. Strains were enriched at least four times before the pellicles were struck out onto LBS plates for single colonies. “Biofilm-up” strains were confirmed by growing cultures from the single colonies and testing them in the pellicle assay.

### Tn mutagenesis and identification of *zur*

To search for putative regulators of the *hnoX* promoter, strain KV10506, which contains a fusion of the *hnoX* promoter to promoterless *lacZ*, was used as a recipient in conjugations with *E. coli* strains that delivered the mariner Tn plasmid pMar-VF1 (16). Tn mutants were selected on LBS plates that contained Erm and X-gal. The resulting colonies were screened for those with increased blue colony color. Colonies that appeared to have increased blue color after the initial screen and subsequent passaging on plates that contained X-gal were collected. Genomic DNA was extracted and used to reintroduce the Tn into a *tfoX*-expressing derivative of the original strain. For those strains that retained the increased blue color, the genomic DNA (gDNA) was also used for semi-arbitrary PCR as described previously (83) with two sequential reactions using primer pairs Arb1 and MJM-440 and Arb2 and MJM-477, respectively (Table S3), followed by product purification using the Clean and Concentrator kit (Zymo Research) and submission to ACGT for sequencing with primer Mariner Tn P2 Lib-PCR.

### Pellicle growth and quantification

*V. fischeri* was grown overnight in LBS liquid media at 24°C with shaking, then the optical densities at 600 nm (OD_600_) were determined. Cells were inoculated to a final OD_600_ of 0.02 into the inner wells of a 24-well microtiter plate that contained 2 mL of LBS with 10 mM CaCl_2_. One overnight culture was inoculated into three separate wells. The plates were then incubated at 24°C for 72 h to allow pellicles to form on the surface of the culture medium. The pellicles were imaged before and after being disturbed with a sterile toothpick using a Zeiss Stemi 2000-c dissecting microscope at a magnification of 6.5×. Images are representative of at least three independent experiments.

To quantify pellicle stickiness, the images of the disrupted pellicles were deidentified and randomized for blinded scoring by a coauthor. The deidentified images were ranked on a scale of 1–4. A ranking of 1 represented no stickiness, 2 represented minor stickiness with the majority of the pellicle unable to be pulled along with the toothpick, 3 represented a sticky pellicle that had minor portions that were not fully cohesive with the rest of the pellicle, and 4 represented full cohesion of the pellicle to itself with architecture. Half-values were given if the phenotypes fell between two rankings. Statistical analyses were performed using a one-way analysis of variance (ANOVA) and Tukey’s multiple comparisons test.

### Whole-genome sequencing

gDNA was extracted from the enriched strains following pellicle enrichment using the Quick-DNA Miniprep Plus Kit (Zymo Research). The resulting gDNA was sequenced by SeqCoast Genomics (Portsmouth, NH, USA). Briefly, samples were prepared for whole-genome sequencing on the Illumina NextSeq2000 using the Illumina DNA Prep tagmentation kit. Sequencing was performed using a 300-cycle flow cell kit for 2 × 150 bp paired reads. PhiX control (1%–2%) was spiked into the run for optimal base calling. DRAGEN v3.10.11 was used to demultiplex and trim reads and run analytics. FastQC metrics were used for quality control (84). Variant calling was done using the Breseq software v0.37.0. Reads were mapped to the *V. fischeri* ES114 reference genome sequences NC_006840.2, NC_006841.2, and NC_006842.1, and mutations were identified (85, 86).

### β-galactosidase activity assay

*V. fischeri* strains that contained a P*hnoX-lacZ* reporter were grown in 5 mL LBS at 24°C with shaking. The cultures were then subcultured 1:100 into fresh 20 mL LBS + 10 mM CaCl_2_. In the case of experiments that included ZnCl_2_, no CaCl_2_ was added. Following growth with shaking at 24°C for 22 h, the OD_600_ of each culture was measured, and a 2 mL sample was collected. Cells were spun down and resuspended in *Z*-buffer (87), and chloroform was added to release β-galactosidase. Fresh ortho-nitrophenyl-β-galactopyranoside (ONPG) was added to start the β-galactosidase reaction, and 1 M NaCO_3_ was added to stop the reaction once samples were sufficiently yellow. To determine β-galactosidase activity, aliquots were transferred to a 96-well plate, and absorbances at 420 and 550 nm were measured on the Biotek Synergy H1 microplate reader. Miller units were calculated following the equation as described (11, 87). Experiments were performed at least three independent times. Statistical analyses were performed using one-way ANOVA and Tukey’s multiple comparisons tests.

### 5' RACE

*V. fischeri* strains were grown overnight in LBS and subcultured the next day in 20 mL of LBS with 10 mM CaCl_2_ at a dilution of 1:100. The strains were grown for 8 h, at which point a 5 mL aliquot of each culture was mixed with 10 mL of RNAprotect (Qiagen). Then, the cells were pelleted, and RNA was extracted from the pellet using the Quick-RNA Miniprep Kit (Zymo Research). Using the extracted RNA, the 5' RACE System for Rapid Amplification of cDNA Ends, version 2.0 (Invitrogen) was used to amplify and purify cDNA, tail it with a dC tail, and amplify the dC-tailed cDNA using provided primers and gene-specific primers 4382, 4383, 4384, and 4424 (Table S3). Of note, the sample obtained after cDNA purification was allowed to evaporate to concentrate the sample further, and the entire sample was used for the dC tailing reaction. Lastly, nested amplification of the cDNA was performed until distinct bands were visible at the right size on agarose gels. These samples were then sequenced with primer 4442 to determine the transcriptional start site.

### Shaking liquid biofilm assay

Strains were grown in 5 mL tTBS overnight at 28°C, then inoculated to an OD_600_ of 0.05 in triplicate into 2 mL of tTBS with 10 mM CaCl_2_ in 13 × 100 mm test tubes. Tubes were incubated at 24°C for 24 h with shaking. Biofilm images were taken using an iPhone camera. The turbidity of the liquid in each tube was measured via OD_600_, ensuring no biofilm was disturbed, to estimate the overall biofilm formation of the sample, which was expected to be inversely correlated with culture OD. Images are representative of at least three independent experiments. Statistical analyses were performed using one-way ANOVA and Tukey’s multiple comparisons tests.

### Wrinkled colony assay

Strains of *V. fischeri* were grown overnight in 5 mL LBS at 28°C with shaking, then subcultured 1:100 into fresh LBS and incubated for approximately 2 h at 28°C with shaking. Samples were normalized to an OD_600_ of 0.2, and aliquots (10 µL) were spotted in triplicate onto 1-day-old LBS plates lacking or containing 10 mM CaCl_2_. These plates were incubated at 24°C for 72 h. The wrinkled colonies were imaged on a Zeiss Stemi 2000-c dissecting microscope with a magnification of 0.8× before and after disruption with a sterile stick. All photos were cropped similarly to allow for accurate comparison. Images are representative of at least three independent experiments.

### Competitive colonization assay

Squid experiments were conducted in accordance with a protocol (LU#218613) approved by Loyola University Chicago’s IACUC. Juvenile *E. scolopes* were collected within ~30 min from the time of hatching and kept in uninoculated saltwater, made using CoralPro salts, to prevent unwanted colonization. Animals that hatched from a single clutch of eggs were kept together for competition experiments. Approximately five squid were placed in fresh seawater that lacked *V. fischeri* to serve as aposymbiotic controls. For the competition assay, multiple animals were introduced into a bowl of seawater that contained the competing strains. *V. fischeri* strains KV10822 and KV9931 were used in the competition experiment; the two strains were distinguished either by the presence of an antibiotic resistance cassette (Erm^R^) in KV10822 or by the distinct colony morphology of the C/A-*hahK*-containing strain (e.g., like those in Fig. 6D). *V. fischeri* strains used in the competition were grown in SWT at 28°C with shaking for 4 h, then were normalized, diluted, and introduced into seawater in a 1:1 ratio. The ratio of strains in the inoculum seawater was subsequently determined by plating onto LBS media and assessing either colony morphology or by subsequently patching the resulting colonies onto Erm-containing plates and scoring for Erm^R^. The squid were incubated at room temperature for 24 h, after which time, the luminescence of each squid was measured to estimate colonization levels. The squid were then euthanized in seawater containing 3% ethanol and frozen at −80°C, then homogenized in sterile seawater to release the bacteria from the light organ. Dilutions of homogenates in sterile seawater were plated onto LBS or LBS + Erm and incubated at 28°C for 48 h. The number of each strain inside each squid was determined using Erm^R^ and/or colony morphology, and the total bacteria per squid were calculated.

### Sequence alignment and phylogenetic analysis

Sequencing reads for the intergenic region between *VF_A0070* and *hnoX*, generated after PCR amplification with primers 4535 and 4441, were aligned using Clustal Omega, a multiple sequence alignment tool embedded in Jalview sequence editor (88). In addition, we included sequences from H905 (reference genome CP160630.1) (54). The phylogenetic trees were generated using NGPhylogeny.fr (89). The embedded PhyML workflow (90) uses the maximum likelihood method to determine phylogeny. The evolutionary model used was General Time Reversible, and the statistical test was a bootstrap of 1,000 iterations.

## Data Availability

Whole-genome sequencing results for KV10708 are available in the National Center for Biotechnology Information’s (NCBI’s) Sequence Read Archive (SRA) under BioProject ID PRJNA1241465.

## SUPPLEMENTAL MATERIAL

Supplemental figures and tables jb.00131-25-s0001.pdf Fig. S1 to S5 and Tables S1 to S3.

ASM does not own the copyrights to Supplemental Material that may be linked to, or accessed through, an article. The authors have granted ASM a non-exclusive, world-wide license to publish the Supplemental Material files. Please contact the corresponding author directly for reuse.
