# Supplementary material for: A single point mutation is sufficient to drive syp-dependent biofilm formation and promote colonization by Vibrio fischeri
Source: J Bacteriol. 2025 Jul 14;207(8):e00131-25. doi: 10.1128/jb.00131-25 (PMC12369376; doi:10.1128/jb.00131-25)
Supplement: Supplemental figures and tables — Fig. S1 to S5 and Tables S1 to S3. [file jb.00131-25-s0001.pdf]

A single point mutation is sufficient to drive *syp*-dependent biofilm formation and promote colonization by *Vibrio fischeri*

Brittany L. Fung[1], Elizabeth G. Musto[1], Linsey Kathure Mugambi[1], Madison L. Lange[1], Jovanka Tepavčević[2], and Karen L. Visick[1,3]

[1]Department of Microbiology and Immunology, Loyola University Chicago, Maywood, IL USA

[2]Department of Biological and Health Sciences, Wheaton College, Wheaton, IL USA

[3]Corresponding author: kvisick@luc.edu

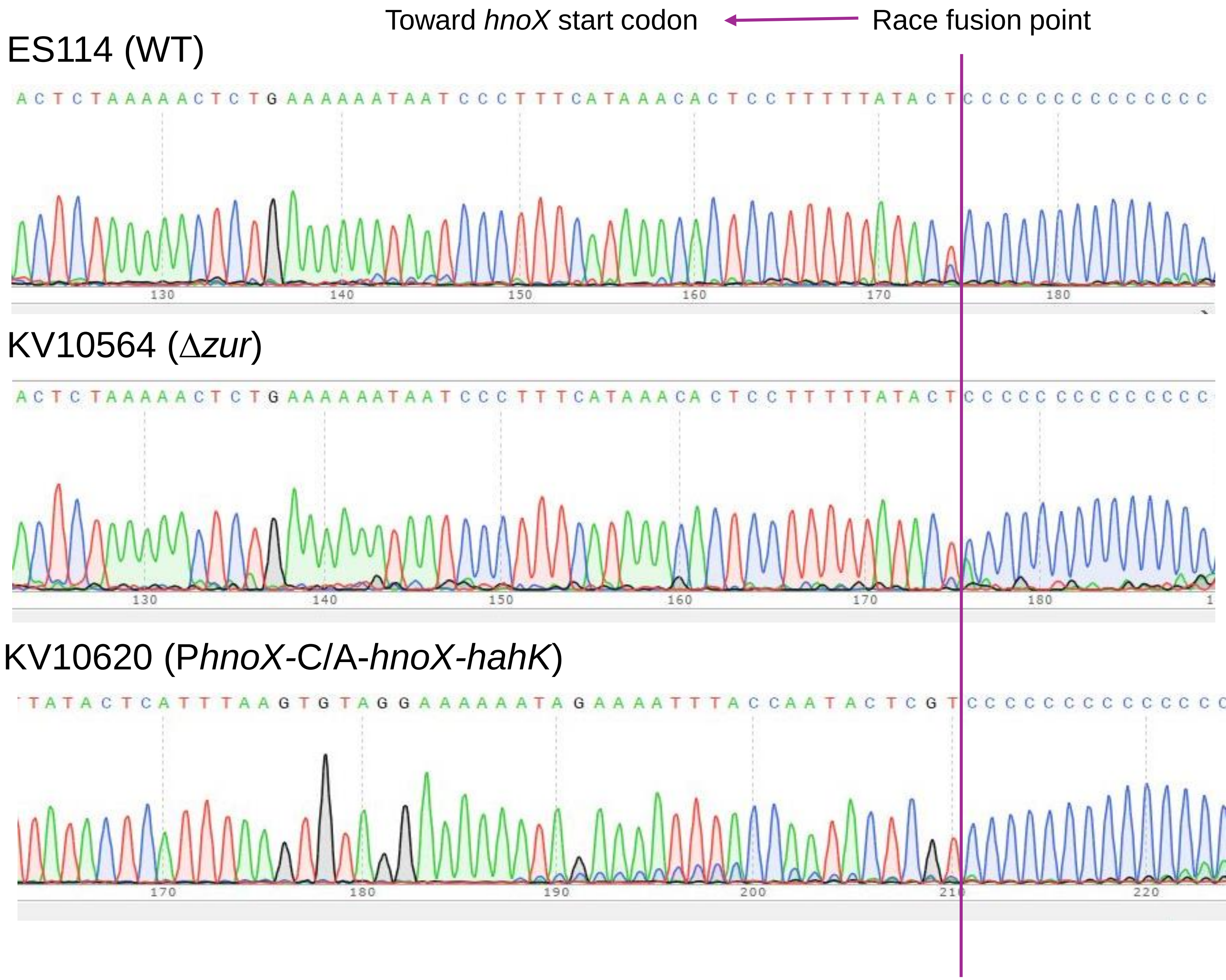

Double stranded promoter region:

GTACGAGTA**TTGGTA**AATTTTCTATTTTTTTCC**TACACT**TAAATGAGTATAAAAAGGAGTGTTT**ATG**
CATGCTCATAACCATTTAAAAGATAAAAAAAGGATGTGAATTTACTCATATTTTTCCTCACAAATAC

Read bottom strand sequence →

Figure S1. 5´ RACE identifies a distinct start site of transcription with the presence of the C/A mutation. 5´ RACE was performed using ES114 (WT), KV10564 (Δ*zur*), and KV10620 (P*hnoX*-C/A-*hnoX*-*hahK*, yielding the traces shown. The string of C nucleotides to the right of the vertical purple line are non-native sequences deriving from the primer used in the reaction. The base immediately to the left of the line indicates the +1 of transcription, and sequences further to the left indicate transcribed bases leading to the *hnoX* coding sequence. At the bottom, the relevant region of the promoter region is shown as a double stranded sequence and contains the identified starts of transcription bolded and underlined in purple (ES114 and KV10564) and green (KV10620), with the predicted wild-type promoter shown in black bolded letters, the putative ribosome binding site underlined in purple, and the ATG start codon underlined in black bolded and underlined letters.

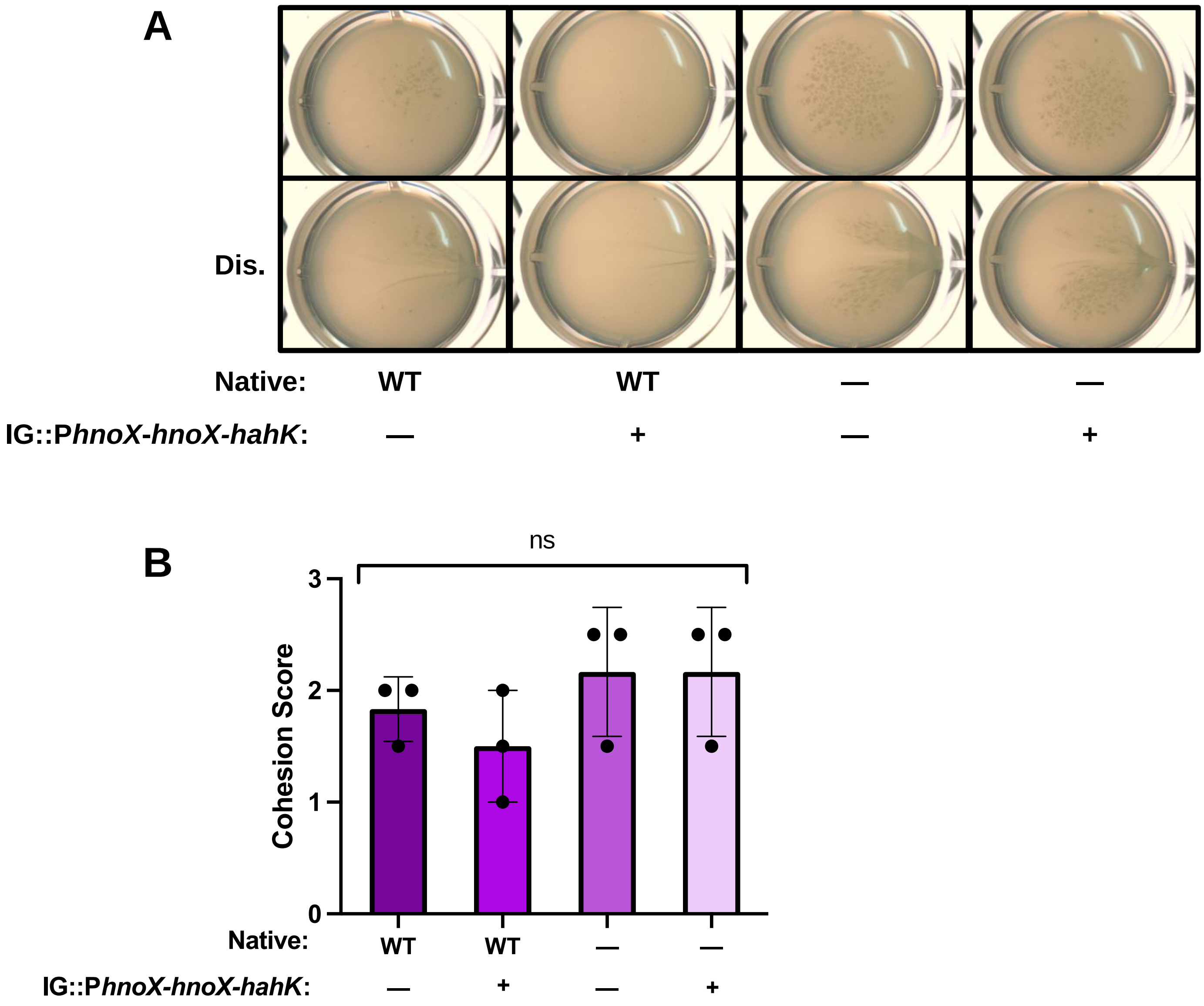

Figure S2. Cohesive biofilm formation is unaffected by an additional copy of the *hnoX-hahK* locus. (A) Representative images of pellicles produced by wild-type strain ES114 (WT/—), a derivative with a second copy of the *hnoX-hahK* operon at a non-native locus (WT/+; KV10653), a Δ(*hnoX-hahK*) mutant (—/—; KV10511), and its derivative that carried, at a non-native site, the wild-type *hnoX-hahK* operon (—/+; KV10513). Cells were grown statically for 72 h in LBS + 10 mM Ca^{2+}. Top, undisturbed; bottom, disturbed with a toothpick (Dis.). Arrows point to areas where sticky biofilm can be visualized. (B) Biofilm cohesion scores of pellicles similar to and including those shown in panel A. ns, not significant. Error bars represent SD.

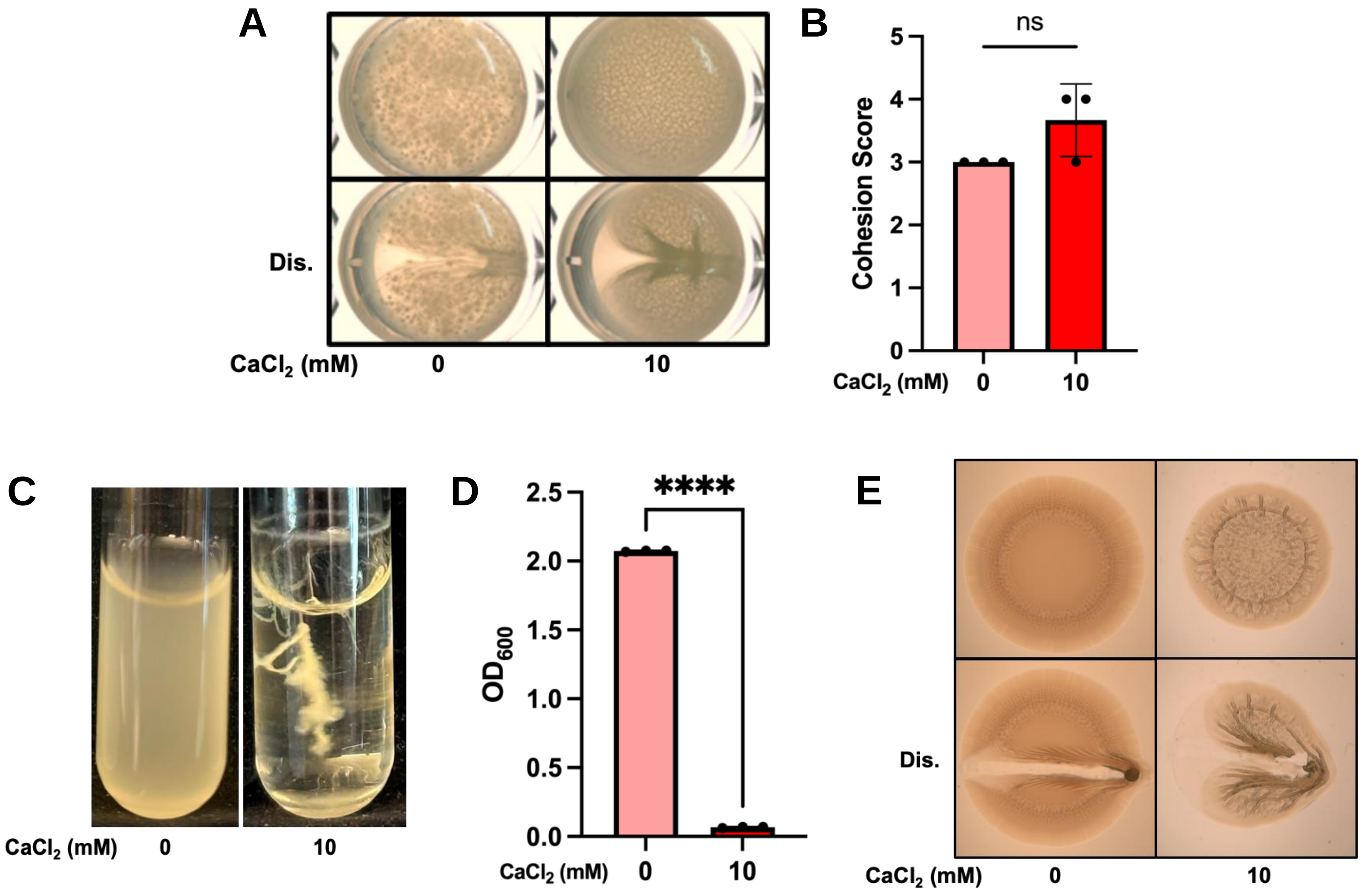

Supplemental Fig. S3. Impact of calcium on phenotypes. For all panels, the strain tested was KV10620 (Δ(*hnoX-hahK*) P*hnoX*-C/A-*hnoX-hahK*. (A) Representative pellicle images produced by strains grown statically for 72 h in LBS with or without 10 mM Ca^{2+}. Top, undisturbed; Bottom, disturbed with a toothpick (Dis.). Arrows point to areas where sticky biofilm can be visualized. (B) Biofilm cohesion scores of pellicles similar to and including those shown in panel A. ns, not significant. Error bars represent SD. (C) Representative images of biofilms formed by cells grown for 24 h under shaking conditions in tTBS with or without 10 mM Ca^{2+}. (D) OD_{600} measurements of the liquid surrounding the biofilm in panel C. ****, P ≤0.0001. Error bars represent SD. (E) Representative images of biofilms formed by cells grown on solid agar LBS plates with or without 10 mM Ca^{2+} and incubated for 72 h at 24°C. Top, undisturbed; bottom, disturbed with a toothpick (Dis.).

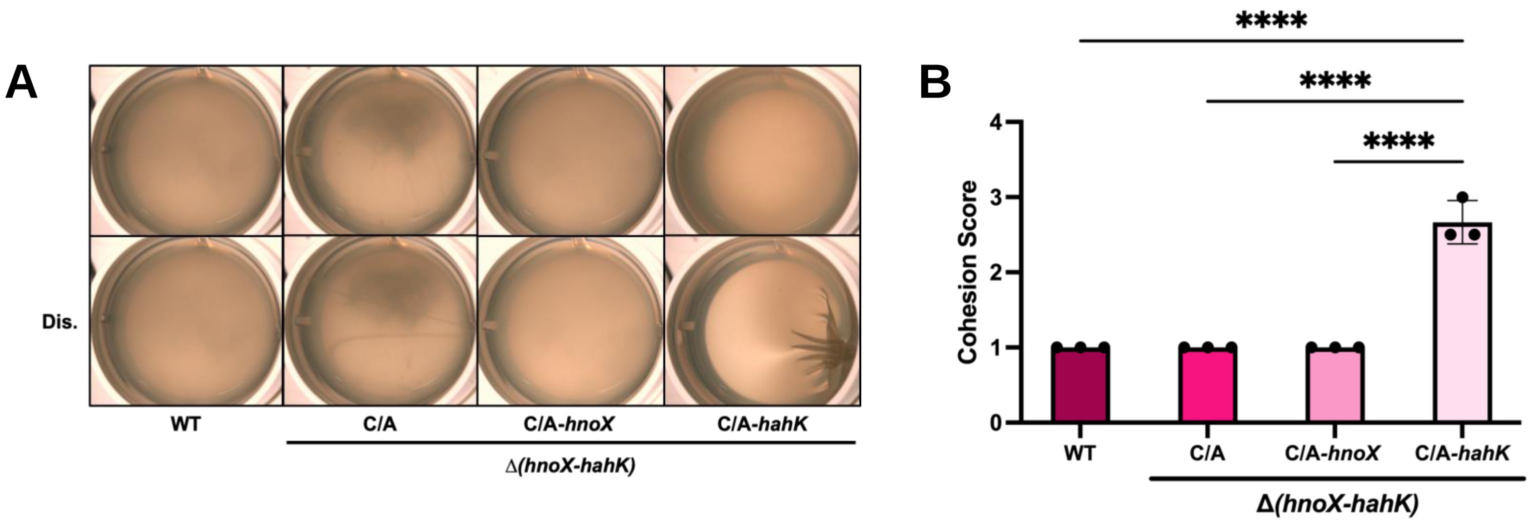

Figure S4. P*hnoX*-C/A-*hahK* produces precocious cohesive pellicles. (A) Representative images of pellicles formed by strains grown statically for 24 h at 24°C in LBS + 10 mM Ca^{2+}. Top, undisturbed; bottom, disturbed with a toothpick (Dis.). The strains evaluated include ES114 (WT) and the Δ(*hnoX-hahK*) mutant carrying either P*hnoX*-C/A-*hnoX-hahK* (C/A; KV10620), P*hnoX*-C/A-*hnoX* (C/A-*hnoX*; KV10711), or P*hnoX*-C/A-*hahK* (C/A-*hahK*; KV10770). (B) Biofilm cohesion score for pellicles similar to and including those shown in panel A. ****, P <0.0001. Error bars represent SD.

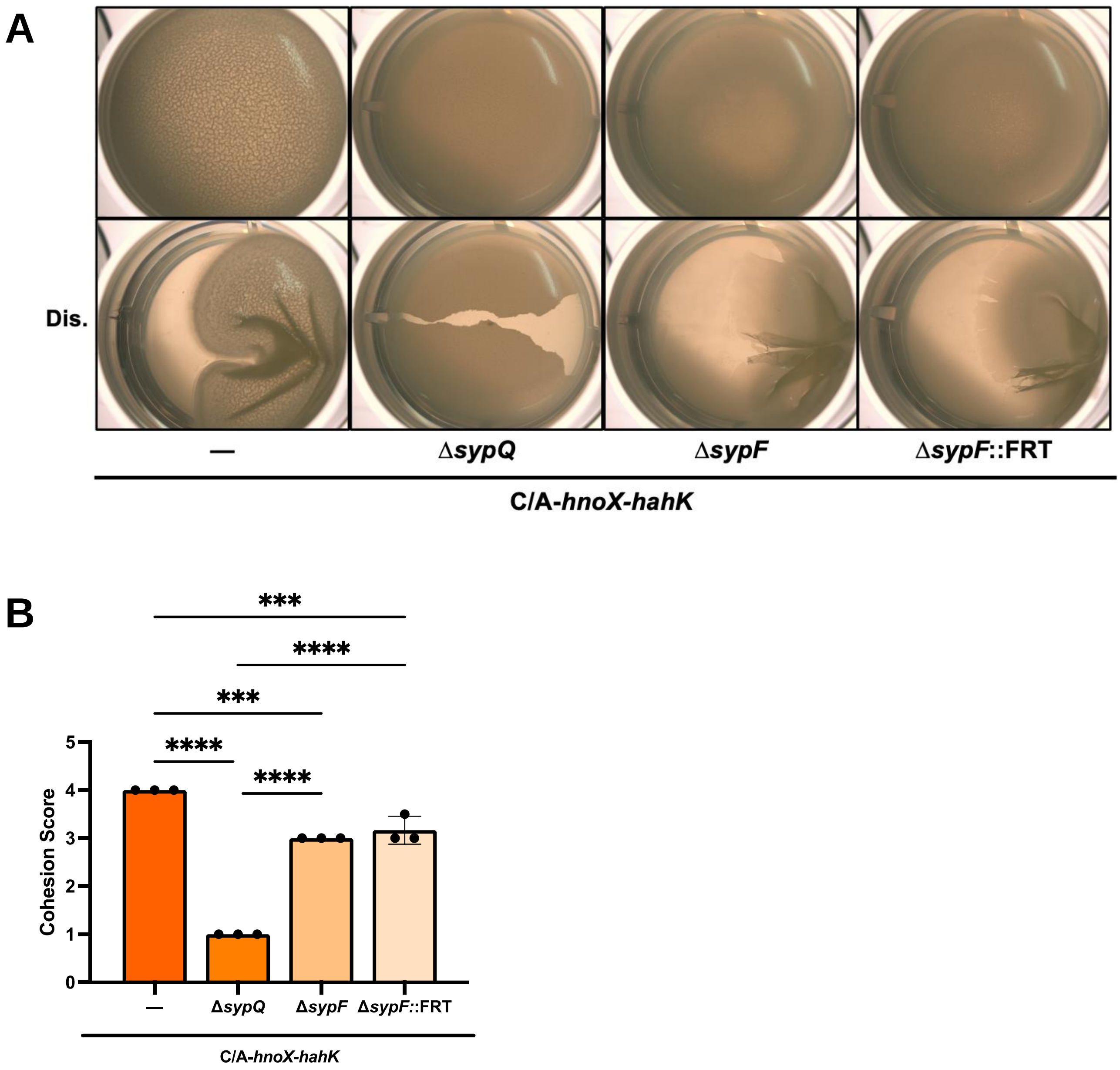

Figure S5. P*hnoX*-C/A-*hnoX*-*hahK* strains form pellicles in absence of SypF. (A) Representative images of pellicles produced by strains that carry a second copy of the P*hnoX*-C/A-*hnoX*-*hahK* operon (C/A-*hnoX*-*hahK*) at a non-native locus in otherwise wild-type (—; KV10701), Δ*sypQ* (KV10700), Δ*sypF* (KV10841), or Δ*sypF::FRT* (KV10833) backgrounds. Cells were grown statically for 72 h in LBS + 10 mM Ca^{2+}. Top, undisturbed; bottom, disturbed with a toothpick (Dis.). (B) Biofilm cohesion scores for pellicles similar to and including those in panel A. ***, P ≤0.001; ****, P ≤0.0001. Error bars represent SD.

Table S1. Strains constructed or used in construction for this study

Name	Genotype[1]	Derivation[2]	Reference
BF450	Δ*sypQ*::FRT IG::P*hnoX-lacZ*	KV10642 + flippase	This study
BF451	Δ*sypQ*::FRT IG::P*hnoX-lacZ* Δ*litR*::FRT	TT KV10642 with gKV9740 (Δ*litR*::FRT-Spec^{R}) + flippase	This study
BF473	ΔsypQ::FRT IG (Erm^{R})::P*hnoX* (C/A)-*lacZ*	TT KV9895 with gKV10447	This study
BF478	Δ*sypQ*::FRT IG::P*hnoX* (C/A)-*lacZ*	BF473 + flippase	This study
KV1787	Δ*sypG*	N/A	(1)
KV3378	Δ*rscS*	N/A	(2)
KV5367	Δ*sypF*	N/A	(3)
KV6351	Δ*rscS* Δ*sypF*	N/A	(3)
KV7371	IG::P*sypA-lacZ*	N/A	(3)
KV8135	IG (Erm^{R})::P*hnoX-hnoX*-HA	N/A	(4)
KV8232	IG::Erm^{R}-trunc Trim^{R}	N/A	(5)
KV8237	IG (Erm^{R})::P*nrdR-hahK*-HA	N/A	(4)
KV8242	Δ*sypF*::FRT-Erm^{R}	N/A	(6)
KV8484	Δ(*hnoX-hahK*)::FRT-Erm^{R}	N/A	(4)
KV9345	Δ*qrr1*::FRT-Trim^{R}	TT ES114 with SP (2424 & 2425 (ES114), 2089 & 2090 (pMLC2), and 2426 & 2427 (ES114))	This study
KV9740	Δ*litR*::FRT-Spec^{R}	N/A	(6)
KV9895	Δ*sypQ*::FRT	N/A	(6)
KV9931	Δ*sypF*::FRT	KV8242 + flippase	This study
KV10050	IG (Erm^{R})::P*litR-litR*	N/A	(6)
KV10408	IG (Erm^{R})::P*hnoX-lacZ*[3]	TT KV7371 with SP ((2185 & 2090 (pKV502), 4255 & 4256 (ES114) and 2822 & 2876 (pKV503))	This study
KV10447	IG (Erm^{R})::P*hnoX* (C/A)-*lacZ*	TT KV7371 with SP (2290 & 4258 (KV10408) and 4257 & 2876 (KV10408))	This study
KV10506	IG::P*hnoX-lacZ*	KV10408 + flippase	This study
KV10508	IG (Erm^{R})::P*hnoX-hnoX-hahK*-HA	TT KV10608 with SP ((2185 & 4278 (KV8135) and 4277 & 2158 (ES114))	This study
KV10511	Δ(*hnoX-hahK*)::FRT	KV8484 + flippase	This study
KV10512	Δ(*hnoX-hahK*)::FRT IG (Erm^{R})::P*hnoX-hnoX-hahK*-HA	TT KV10511 with gKV10508	This study
KV10513	Δ(*hnoX-hahK*)::FRT IG::P*hnoX-hnoX-hahK*-HA	KV10512 + flippase	This study
KV10514	Δ(*hnoX-hahK*)::FRT IG (Erm^{R})::P*hnoX* (C/A)-*hnoX-hahK*-HA	TT KV10513 with SP ((2185 & 4258 (KV10508) and 4257 & 4278 (KV10508))	This study
KV10519	IG::P*hnoX-lacZ zur*::Tn-Mariner	Conjugation KV10506 with pMar-VF1	This study
KV10528	*zur*::Tn-Mariner	NT ES114 with gKV10519	This study
KV10549	Δ*zur*::FRT-Spec^{R}	NT ES114 with SP (4333 & 4334 (ES114) + 2089 & 2090 (pKV534) and 4335 & 4336 (ES114)	This study
KV10552	Δ*zur*::FRT-Erm^{R}	TT ES114 with SP (4333 & 4334 (ES114), 2089 & 2090 (pKV494), and 4335 & 4336 (ES114))	This study
KV10564	Δ*zur*::FRT	KV10549 + flippase	This study
KV10608	IG::P*nrdR-hahK*-HA	KV8237 + flippase	This study
KV10620	Δ(*hnoX-hahK*)::FRT IG::P*hnoX* (C/A)-*hnoX-hahK*-HA	KV10514 + flippase	This study
KV10621	Δ*sypQ*::FRT IG (Erm^{R})::P*hnoX* (C/G)-*lacZ*	TT KV9895 with SP (2185 & 4369 (gKV10408) and 4368 & 2876 (gKV10408))	This study
KV10622	Δ*sypQ*::FRT IG (Erm^{R})::P*hnoX* (C/T)-*lacZ*	TT KV9895 with gDNA from intermediate strain (NT KV7371 with SP (2185 & 4371 (gKV10408) and 4370 & 2876 (gKV10408))	This study
KV10624	Δ*sypQ*::FRT IG (Erm^{R})::P*hnoX*-Trunc-1-*lacZ*	TT KV9895 with gDNA from intermediate strain with SP (NT KV7371 (2185 & 2090 (gKV10408) and 4366 & 2876 (gKV10408))	This study

KV10625	Δ*sypQ*::FRT IG (Erm^R)::P*hnoX*-Trunc-2-*lacZ*	TT KV9895 with gDNA from intermediate strain (NT KV7371 with SP (2185 & 2090 (gKV10408) and 4367 & 2876 (gKV10408))	This study
KV10626	Δ*sypF* IG (Erm^R)::P*hnoX* (C/A)-*hnoX-hahK*-HA	TT KV5367 with gKV10514	This study
KV10627	Δ*rscS* IG (Erm^R)::P*hnoX* (C/A)-*hnoX-hahK*-HA	TT KV3378 with gKV10514	This study
KV10628	Δ*sypG* IG (Erm^R)::P*hnoX* (C/A)-*hnoX-hahK*-HA	TT KV1787 with gKV10514	This study
KV10640	Δ*sypQ*::FRT IG (Erm^R)::P*hnoX* (C/A)-*hnoX-hahK*-HA	TT KV9895 + gKV10514	This study
KV10642	Δ*sypQ*::FRT IG (Erm^R)::P*hnoX-lacZ*	TT KV9895 with gKV10408	This study
KV10645	Δ*sypQ*::FRT IG::P*hnoX* (C/G)-*lacZ*	KV10621 + flippase	This study
KV10646	Δ*sypQ*::FRT IG::P*hnoX* (C/T)-*lacZ*	KV10622 + flippase	This study
KV10648	Δ*sypQ*::FRT IG::P*hnoX*-Trunc-2-*lacZ*	KV10624 + flippase	This study
KV10649	Δ*sypQ*::FRT IG::P*hnoX*-Trunc-3-*lacZ*	KV10625 + flippase	This study
KV10650	Δ*sypF* IG::P*hnoX* (C/A)-*hnoX-hahK*-HA	KV10626 + flippase	This study
KV10650	Δ*sypF* IG::P*hnoX* (C/A)-*hnoX-hahK*-HA	KV10626 + flippase	This study
KV10651	Δ*rscS* IG::P*hnoX* (C/A)-*hnoX-hahK*-HA	KV10627 + flippase	This study
KV10652	Δ*sypG* IG::P*hnoX* (C/A)-*hnoX-hahK*-HA	KV10628 + flippase	This study
KV10653	IG::P*hnoX-hnoX-hahK*-HA	KV10508 + flippase	This study
KV10661	Δ*sypF* IG::P*hnoX* (C/A)-*hnoX-hahK*-HA attTn*7*::*sypF*-flag	KV10650 with Tn*7* insertion from pANN20	This study
KV10662	Δ*sypF* IG::P*hnoX* (C/A)-*hnoX-hahK*-HA attTn*7*::*sypF*-Hpt-flag	KV10650 with Tn*7* insertion from pANN50	This study
KV10663	Δ*sypG* IG::P*hnoX* (C/A)-*hnoX-hahK*-HA attTn*7*::*sypG*-flag	KV10652 with Tn*7* insertion from pSST5	This study
KV10665	IG (Erm^R)::P*hnoX* (C/A)-*hnoX-hahK*-HA	TT ES114 with gKV10514	This study
KV10666	Δ*sypQ*::FRT IG::P*hnoX-lacZ* Δ*qrr1*::FRT-Trim^R	TT BF450 with gKV9345	This study
KV10668	Δ*sypQ*::FRT IG::P*hnoX-lacZ* Δ*zur*::FRT-Erm^R	TT BF450 with gKV10552	This study
KV10696	Δ*sypQ*::FRT IG :::P*hnoX-lacZ zur*::Tn-Mariner	TT BF450 with gKV10528	This study
KV10697	IG (Erm^R)::P*hnoX* (C/A)-*hnoX*-HA	TT ES114 with SP (2185 & 4258 (gKV8135) & 4257 & 1487 (gKV8135))	This study
KV10700	Δ*sypQ*::FRT IG::P*hnoX* (C/A)-*hnoX-hahK*-HA	KV10640 + flippase	This study
KV10701	IG::P*hnoX* (C/A)-*hnoX-hahK*-HA	KV10665 + flippase	This study
KV10704	Δ(*hnoX-hahK*)::FRT IG (Erm^R)::P*hnoX* (C/A)-*hnoX*-HA	TT KV10511 with gKV10697	This study
KV10708	IG (Erm^R)::p*litR-litR* + C74297A (and other possible changes)	Increased biofilm suppressor of KV10050	This study
KV10711	Δ(hnoX-hahK)::FRT IG::P*hnoX* (C/A)-*hnoX*-HA	KV10704 + flippase	This study
KV10719	Δ*rscS* Δ*sypF* IG (Erm^R)::P*hnoX* (C/A)-*hnoX-hahK*-HA	TT KV6351 with gKV10514	This study
KV10762	Δ*sypQ*::FRT IG (Erm^R)::P*hnoX*-Trunc-3-*lacZ*	TT KV9895 with gDNA from intermediate derived from NT KV7371 with SP (2185 & 2090 (pKV502) & 4414 & 2876 (gKV10408))	This study
KV10767	IG (Erm^R)::P*hnoX* (C/A)-*hahK*-HA	TT KV10608 with SP (2185 & 4374 and 4375 & 2158, amp from intermediate strain generated by NT of KV10608 with SP (2185 & 4374 (gKV10514) and 4375 & 2158 (gKV10514))	This study
KV10768	Δ(*hnoX-hahK*)::FRT IG (Erm^R)::P*hnoX* (C/A)-*hahK*-HA	TT KV10511 with gKV10767	This study
KV10770	Δ(*hnoX-hahK*)::FRT IG::P*hnoX* (C/A)-*hahK*-HA	KV10768 + flippase	This study
KV10772	Δ*sypQ*::FRT IG::P*hnoX*-Trunc-3-*lacZ*	KV10762 + flippase	This study
KV10808	Δ*sypF* IG (Erm^R)::P*hnoX* (C/A)-*hnoX-hahK*-HA	TT KV5367 with gKV10514	This study
KV10809	ΔsypF IG (Erm^R)::P*hnoX* (C/A)-*hahK*-HA	TT KV5367 with gKV10768	This study
KV10821	Δ*sypF*::FRT IG (Erm^R)::P*hnoX* (C/A)-*hnoX-hahK*-HA	TT KV9931 with gKV10514	This study
KV10822	Δ*sypF*::FRT IG (Erm^R)::P*hnoX* (C/A)-*hahK*-HA	TT KV9931 with gKV10768	This study
KV10826	IG (Erm^R)::P*hnoX* (C/A)-*hahK*-DA-HA	TT KV8232 with SP (2290 & 2179 (KV10768) and 2178 & 1487 (KV10768))	This study
KV10829	Δ*sypF* IG::P*hnoX* (C/A)-*hahK*-HA	KV10809 + flippase	This study
KV10833	Δ*sypF*::FRT IG::P*hnoX* (C/A)-*hnoX-hahK*-HA	KV10821 + flippase	This study
KV10837	Δ*rscS* Δ*sypF* IG::P*hnoX* (C/A)-*hnoX-hahK*-HA	KV10719 + flippase	This study
KV10838	IG::P*hnoX* (C/A)-*hahK*-HA	KV10767 + flippase	This study
KV10841	Δ*sypF* IG::P*hnoX* (C/A)-*hnoX-hahK*-HA	KV10808 + flippase	This study

KV10846	Δ*luxU*::FRT-Spec^R	TT ES114 with SP (4476 & 4477 (ES114), 2089 & 2090 (pKV534), and 4478 & 4479 (ES114))	This study
KV10933	IG::P*hnoX* (C/A)-*hahK*-DA-HA	KV10826 + flippase	This study
KV10962	Δ*sypF* IG::P*hnoX* (C/A)-*hnoX-hahK*-HA Δ*luxU*::FRT-Spec^R	TT KV10650 with gKV10846	This study
KV10963	IG::P*hnoX* (C/A)-*hnoX-hahK*-HA Δ*luxU*::FRT-Spec^R	TT KV10701 with gKV10846	This study

[1]Genotype abbreviations are as follows: Intergenic region (IG) between the genes *yeiR* and *glmS*, generated using FRT-flanked Erm^R, IG (Erm^R) (shorthand for IG (*yeiR*-FRT-Erm^R/*glmS*)), derivative in which the Erm^R cassette has been removed, IG (shorthand for IG (*yeiR*-FRT/*glmS*)); attTn*7*, insertion of Tn*7* at the Tn*7 att* site between *yeiR* and *glmS*; C/A, C74297A, the point mutation upstream of the *hnoX* promoter, and correspondingly, C/T and C/G changes; HA, hemagglutinin epitope tag; flag, flag epitope tag; DA, aspartate to glutamate substitution; Trunc, truncation

[2]Derivation abbreviations are as follows: N/A, not applicable, published strain, not derived for this work; SP=product derived by Splicing by Overlap extension PCR; TT, TfoX-mediated transformation; + Flippase, indicates flippase encoded by pKV496 delivered by conjugation to resolve an antibiotic resistance cassette

[3]In the construction of this fusion, a point mutation occurred in the linker between the promoter and *lacZ*. All P*hnoX-lacZ* fusion constructs were derived directly or indirectly from this strain, such that they all contained the same base change.

Table S2. Plasmids used in this study

Plasmid	Description	Reference
pEVS107	Tn*7* delivery vector; Kan^R Erm^R	(7)
pMar-VF1	Mariner Tn delivery plasmid	(8)
pANN20	Tn*7-sypF*; Kan^R Erm^R	(3)
pANN50	Tn*7-sypF*-Hpt; Kan^R Erm^R	(3)
pJJC4	plostfoX, *litR*; Cm^R	(9)
pKV496	Flippase plasmid; Kan^R	(5)
pKV502	pJET + *yeiR*-Erm^R, Ap^R, Erm^R	(5)
pKV503	pJET + *glmS*, Ap^R	(5)
pKV534	pJET + FRT-Spec^R, Ap^R, Spec^R	This study
plostfoX	*tfoX*, Cm^R	(10)
pMLC2	pJET + FRT-Trim^R, Ap^R, Trim^R	(5)
pSST5	Tn*7-sypG*; Kan^R Erm^R	This study
pUX-BF13	Tn*7* transposase, Ap^R	(11)

Table S3. Primers used in this study

Primer	Sequence[1]
1487	GGTCGTGGGGAGTTTTATCC
2089	CCATACTTAGTGCGGCCGCCTA
2090	CCATGGCCTTCTAGGCCTATCC
2158	tcgcgccacattgtatttgg
2178	GTTTTAATGGcTTGTCGAATGCCGATTCTTG
2179	CATTCGACAAgCCATTAAAACAATATCAA
2185	CTTGATTTATACAGCGAAGGAG
2196	Tccatacttagtgcggccgccta
2290	AAGAAACCGATACCGTTTACG
2424	GGTATCTTTTGGATTCTCTTGG
2425	taggcggccgcactaagtatggCCTATTGCAGGGAGCGTGCCAAC
2426	ggataggcctagaaggccatggGCTATAAAATCAATAACTAACTATTC
2427	CGCTTAGGTGAGTTTGATGTCC

2822	AGGAAACAGCTATGACCATGATTACGGATTCAC
2876	GAAACGCCGAGTTAACGCC
4255	ggataggcctagaaggccatggTTTTTGATTTTCTGCAAGTTAG
4256	catggtcatagctgttTCCTTTTTATACTCATTTAAGTGTAG
4257	CTTTATTTTTTGATaTCCTTATCTGTACGAGTATTG
4258	CAGATAAGGATaTCAAAAAATAAAGTGCATAAC
4277	CAAGTACAGAGACCGAAATG
4278	GATTTAGTTAAGGTAAAACGAAC
4333	CACCAAAACGGCCATTTTGTAC
4334	taggcggccgcactaagtatggTGTACGATTCAATGAAAATATGTC
4335	ggataggcctagaaggccatggAATAAGAAAAATTAAAATAATATTTAAAGG
4336	ACCTCTATCGTTCTAGTCCC
4366	ggataggcctagaaggccatggTTATTCGAATGTAATTTCAAATCC
4367	ggataggcctagaaggccatggCCTATTTTTATTATCTATTTTGCC
4368	CTTTATTTTTTGATgTCCTTATCTGTACGAGTATTG
4369	CAGATAAGGAcATCAAAAAATAAAGTGCATAAC
4370	CTTTATTTTTTGATtTCCTTATCTGTACGAGTATTG
4371	CAGATAAGGAaATCAAAAAATAAAGTGCATAAC
4374	gttttatcaacatcCATAAACACTCCTTTTTATACTC
4375	ggagtgtttATGGATGTTGATAAAACAATTGAATTATTG
4414	ggataggcctagaaggccatggCTCCTTATCTGTACGAGTATTGG
4382	CGAACTTGAGAACCATCATCTAAAACAGG
4383	GAATATCGATTATCTCATTAAAATGTTCTGC
4384	CCTAAAATCAATCCAAAACAAACATGAGAG
4424	GAGTTAGTTGAAGATAAATTTGGG
4441	CGCCAGTGACTTTACTC
4442	GCTTACGCCAGTGACTTTACTC
4476	CCTCCTCTAAGAGACAGAGG
4477	taggcggccgcactaagtatggCTGACTCACGATAGTCCCC
4478	ggataggcctagaaggccatggACTGAAATGGCTTATCGAGAACTC
4479	CATCCATCAGACTTGGGAGC
4535	GAAGTGCTAATAATGATATGAACG
Mariner Tn P2 Lib-PCR	GGGGACTTATCATCCAACCTGT
MJM-440	TCAACACACTCTTAAGTTTGCTTC
MJM-477	TTCCATAACTTCTTTTACGTTTCC
Arb 1	GGCCACGCGTCGACTAGTACNNNNNNNNNNGATAT
Arb 2	GGCCACGCGTCGACTAGTAC

[1]Lowercase letters represent “tail” sequences not complementary to target sequences

References

1. Hussa EA, O'Shea TM, Darnell CL, Ruby EG, Visick KL. 2007. Two-component response regulators of *Vibrio fischeri*: identification, mutagenesis, and characterization. J Bacteriol 189:5825-38.
2. Geszvain K, Visick KL. 2008. Multiple factors contribute to keeping levels of the symbiosis regulator RscS low. FEMS Microbiol Lett 285:33-9.
3. Norsworthy AN, Visick KL. 2015. Signaling between two interacting sensor kinases promotes biofilms and colonization by a bacterial symbiont. Mol Microbiol 96:233-248.
4. Thompson CM, Marsden AE, Tischler AH, Koo J, Visick KL. 2018. *Vibrio fischeri* biofilm formation prevented by a trio of regulators. Appl Environ Microbiol 84:e01257-18.
5. Visick KL, Hodge-Hanson KM, Tischler AH, Bennett AK, Mastrodomenico V. 2018. Tools for rapid genetic engineering of *Vibrio fischeri*. Appl Environ Microbiol 84:e00850-18.
6. Fung BL, Visick KL. 2025. LitR and its quorum-sensing regulators modulate biofilm formation by *Vibrio fischeri*. J Bacteriol 207:e0047624.
7. McCann J, Stabb EV, Millikan DS, Ruby EG. 2003. Population dynamics of *Vibrio fischeri* during infection of *Euprymna scolopes*. Appl Environ Microbiol 69:5928-34.
8. Brooks JF, 2nd, Gyllborg MC, Cronin DC, Quillin SJ, Mallama CA, Foxall R, Whistler C, Goodman AL, Mandel MJ. 2014. Global discovery of colonization determinants in the squid symbiont *Vibrio fischeri*. Proc Natl Acad Sci U S A 111:17284-9.
9. Cohen JJ, Eichinger SJ, Witte DA, Cook CJ, Fidopiastis PM, Tepavcevic J, Visick KL. 2021. Control of competence in *Vibrio fischeri*. Appl Environ Microbiol 87:e01962-20.
10. Pollack-Berti A, Wollenberg MS, Ruby EG. 2010. Natural transformation of *Vibrio fischeri* requires *tfoX* and *tfoY*. Environ Microbiol 12:2302-11.
11. Bao Y, Lies DP, Fu H, Roberts GP. 1991. An improved Tn*7*-based system for the single-copy insertion of cloned genes into chromosomes of Gram-negative bacteria. Gene 109:167-168.
